# Inhibition of mTORC1 Enhances the Translation of Chikungunya Proteins *via* the Activation of the MnK/eIF4E Pathway

**DOI:** 10.1371/journal.ppat.1005091

**Published:** 2015-08-28

**Authors:** Pierre-Emmanuel Joubert, Kenneth Stapleford, Florence Guivel-Benhassine, Marco Vignuzzi, Olivier Schwartz, Matthew L. Albert

**Affiliations:** 1 Unité Immunobiologie des Cellules Dendritiques, Département d’Immunologie, Institut Pasteur, Paris, Cedex 15, France; 2 INSERM U818, Paris, France; 3 Unité des populations virales et Pathogenèse, Département de Virologie, Institut Pasteur, Paris, Cedex 15, France; 4 Unité Virus et Immunité, Département de Virologie Institut Pasteur, Paris, Cedex 15, France; 5 Centre d’Immunologie Humaine, Département d’Immunologie, Institut Pasteur, Paris, Cedex 15, France; The University of Chicago, UNITED STATES

## Abstract

Chikungunya virus (CHIKV), the causative agent of a major epidemic spanning five continents, is a positive stranded mRNA virus that replicates using the cell’s cap-dependent translation machinery. Despite viral infection inhibiting mTOR, a metabolic sensor controls cap-dependent translation, viral proteins are efficiently translated. Rapalog treatment, silencing of *mtor* or *raptor* genes, but not *rictor*, further enhanced CHIKV infection in culture cells. Using biochemical assays and real time imaging, we demonstrate that this effect is independent of autophagy or type I interferon production. Providing *in vivo* evidence for the relevance of our findings, mice treated with mTORC1 inhibitors exhibited increased lethality and showed a higher sensitivity to CHIKV. A systematic evaluation of the viral life cycle indicated that inhibition of mTORC1 has a specific positive effect on viral proteins, enhancing viral replication by increasing the translation of both structural and nonstructural proteins. Molecular analysis defined a role for phosphatidylinositol-3 kinase (PI3K) and MAP kinase-activated protein kinase (MnKs) activation, leading to the hyper-phosphorylation of eIF4E. Finally, we demonstrated that in the context of CHIKV inhibition of mTORC1, viral replication is prioritized over host translation *via* a similar mechanism. Our study reveals an unexpected bypass pathway by which CHIKV protein translation overcomes viral induced mTORC1 inhibition.

## Author Summary

The ongoing chikungunya epidemic outbreak in the Caribbean, Central and South America highlights how poor is our understanding of CHIKV pathogenesis and the urgent need for new strategies that may limit CHIKV spread. Immunological studies have suggested that dissemination of infection is largely determined by early events of viral-host cell interactions. In our prior study, we investigated the role of type I interferon responses and the autophagy pathway as mediators of viral control. Here, we evaluated the role of mTOR, making the surprising discovery that inhibition of mTORC1 enhances viral protein translation independently of type I IFN and autophagy. While the inhibition of mTORC1 has no impact on viral binding or entry, we observed an increased translation of both structural and nonstructural viral proteins. Interestingly, the positive impact of mTORC1 inhibition is restricted to viral proteins, as compared to host cap-dependent protein translation that remains suppressed. Further analysis demonstrates that this bypass pathway is mediated the activation of PI3K and MnKs, which in turn hyper-phosphorylate eIF4E, a critical initiation protein for translation. Notably, CHIKV replication enables this pathway as a means to efficiently replicate. Thus, our study provides an unexpected role for mTORC1 in the control of CHIKV infection and highlights a new strategy by which the expression of CHIKV proteins can bypass and/or use the inhibition of mTORC1.

## Introduction

Since 2005 there has been a recurrence of Chikungunya disease, with the initial outbreak occurring in the French territory La Réunion [1]. The epidemic has spread worldwide, with outbreaks in five continents [2,3]. Notably, in just nine months during, Chikungunya virus (CHIKV) spread to 22 countries in the Caribbean, Central and South America, resulting in hundreds of thousands of cases [4]. The treatment of CHIKV infections relies on symptomatic relief, as no effective anti-viral agents are available [3]. We therefore set out to investigate cellular pathways that regulate CHIKV replication and spread.

Like other alphaviruses, CHIKV contains a single positive stranded RNA genome of approximately 11.5 kB [5]. The genomic RNA is capped and polyadenylated, and encodes two open reading frames (ORFs). The 5' ORF encodes four nonstructural proteins that participate in genome replication [6]. It is expressed *via* cap-dependent translation as an nsP1–3 or nsP1–4 polyprotein that is cleaved by the nsP2-encoded protease. The structural proteins are encode by a single ORF within the subgenomic region and is also translated *via* a cap-dependent mechanism [7]. As observed for other alpahviruses (e.g., Sindbis), CHIKV infection induces several cell stress responses, which might be associated with pathogenesis [8]. In our previous work, we showed that cells infected by CHIKV exhibit phenotypic characteristics of oxidative stress, endoplasmic reticulum stress, interferon induction, autophagy and apoptosis [9]. Notably, these adaptations are due, in part, to modification of the regulator kinase mTOR.

The mammalian target of Rapamycin (mTOR) coordinates cellular catabolic and anabolic processes to promote growth, proliferation and survival signals [10]. mTOR elicits its pleiotropic functions in the context of two functionally distinct signaling complexes, termed mTOR complex 1 (mTORC1) and complex 2 (mTORC2). mTORC1, which contains mTOR, mLST8/GβL, Raptor and PRAS40, plays a key role in cap-dependent translation initiation by directly phosphorylating p70 S6 kinase 1 (S6K1) and eIF4E-binding protein 1 (4E-BP1), and is sensitive to Rapamycin [10]. S6K1 phosphorylate several proteins that are associated with mRNA translation or its control, including ribosomal protein S6 and eukaryotic initiation factor 4B (eIF4B) [10]. The 4E-BP1 are small phosphoproteins which bind to eIF4E at a site that overlaps its interaction site for eIF4G, preventing the formation of eIF4F complex essential for the initiation of capped mRNA [10]. mTORC2 shares mTOR and mLST8/GβL with mTORC1, but possesses three unique components, namely, rictor, mSin1 and PRR5/Protor [11]. Despite the presence of mTOR, mTORC2 is considerably less susceptible to Rapamycin inhibition [10].

As a master sensor of cellular homeostatic perturbations, several studies have investigated the relationship between mTOR activity and viral infection. Numerous viruses modify the activity of mTOR (or mTOR pathways) [12–14]. Regulation of mTOR induces virus-specific effects that are often with opposing action. For example, blocking of mTOR by Rapamycin or TORISEL (referred to be the class of drugs known as Rapalog) inhibit the replication of HCMV [12]; yet Rapalog treatment facilitates HEV replication [13]. During HCV infection of liver cells, activation of PI3K-PKB-mTOR mediates both viral supportive functions (e.g., prevention of apoptosis in HCV-infected cells), and the production of antiviral interferon [14,15].

Regarding CHIKV infection, we previously observed that transient inhibition of the mTORC1 pathway during the first hour of infection [9]. While we demonstrated that this transient inhibition of mTOR correlates with CHIKV-induced autophagy, the direct role of mTOR activity on CHIKV replication remained unknown. Herein, we report that inhibition of mTORC1 enhances CHIKV replication in a cell-intrinsic manner and promotes *in vivo* spread and worsening of disease. Moreover, we show that mTORC1 impacts CHIKV infection independently of type I IFN production and autophagy. Instead, its actions directly effect translation of viral proteins *via* the activation of the MnK/eIF4E pathway. These results reveals a role for mTORC1 as a host defense mechanism that limits CHIKV replication and highlights a new strategy by which the expression of CHIKV proteins can bypass the inhibition of mTORC1.

## Results

### mTORC1 regulates the magnitude of CHIKV infection

**Fig 1:**
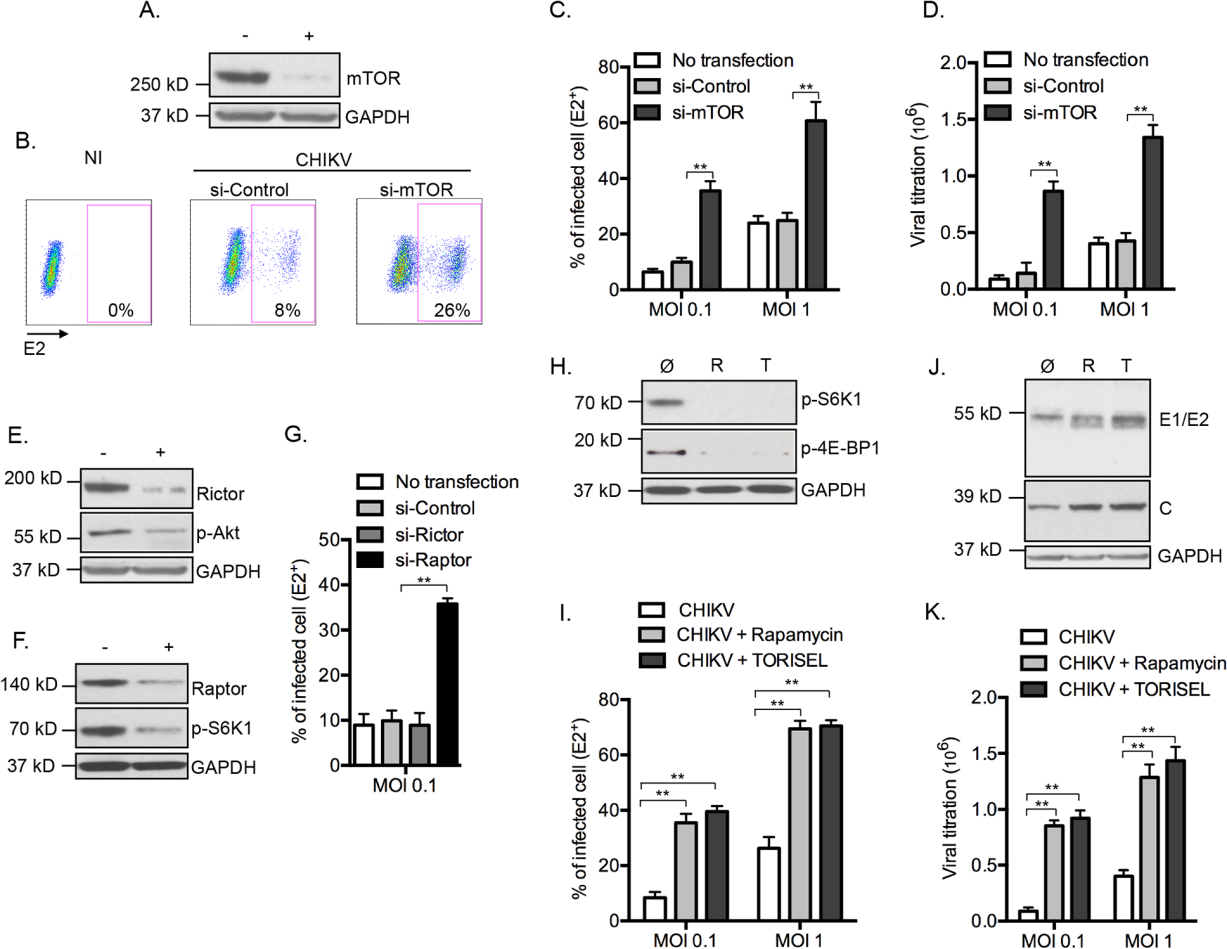
Inhibition of mTORC1 favors CHIKV infection. **(A-D)** MEFs pretreated with *mtor* siRNA for 3d were infected by CHIKV at indicated MOI for 24h. (**A**) Western blot was performed 24h post-infection (p.i.) using anti-mTOR and anti-GAPD antibodies. Similar results were observed in four independent experiments. **(B, C)** The percentage of intracellular E2 staining was analyzed using an anti-E2 antibody, assessed using flow cytometer. Representative FACS data is shown **(B)**, and results shown for MOI 0.1 and 1.0 (**C**). Greater than 10,000 cells were acquired. Bars indicate mean values ±SEM from five independent experiments. **(D)** Extracellular viral titers were determined at 24h p.i. and results were expressed as TCID_50_/ml. Bars indicate mean values ±SEM from four independent experiments. **(E-G)** MEFs pretreated with siRNA targeting *rictor* or *raptor* were infected by CHIKV (MOI = 0.1) for 24h. **(E, F)** Western blot was performed using anti-rictor, anti-raptor and anti-GAPD antibodies. Reduced activity of mTORC1 and mTORC2 was confirmed by following the phosphorylation on tyrosine 389 of S6K-1 (p-S6K1) and the phosphorylation on serine 473 of Akt (p-Akt), respectively. Similar results were observed in three independent experiments. CHIKV protein E2 was stained and the percentage of positive cells was determined and represented as graph **(G)**. Bars indicate mean values ±SEM from three independent experiments. **(H)** MEFs were treated with Rapamycin (100 nM) or TORISEL (0.1 mg/ml) for 24h and **r**educed activity of mTORC1 was confirmed by following the phosphorylation on tyrosine 389 of S6K-1 (pS6K1) and the phosphorylation on serine 65 of 4E-BP1 (p-4E-BP1). Similar results were observed in five independent experiments. **(I-K)** MEFs were infected with CHIKV at indicated MOI for 24h in presence of Rapamycin (100 nM) or TORISEL (0.1 mg/ml). **(I)** The percentage of E2 positive cells is depicted. Bars indicate mean values ±SEM from five independent experiments. **(J)** Western blot was performed using antibodies against envelope proteins 1 and 2 (anti-E1/E2), nucleocapsid (anti-C) and GAPDH. Similar results were observed in two independent experiments. **(K)** Extracellular viral titers were determined at 24h p.i. Results were expressed as TCID_50_/ml. Bars indicate mean values ±SEM from four independent experiments. Student’s test **, P < 0.05. +, indicates si-*mtor* (A), si-*rictor* (E) and si-*raptor* (F);-, si-control; Ø, control buffer for inihibitor experiments; R, Rapamycin; T, TORISEL.

To determine the relationship between CHIKV infection and mTOR activity, mouse embryonic fibroblast (MEFs) cells were transfected with siRNA to suppress *mTOR* expression (Fig 1A), followed by infection with CHIKV (CHIKV-21, the La Réunion 2005 strain). Surprisingly, we observed a 3 fold increase in the number of infected cells as compared to control siRNA treatment (Fig 1B and 1C). Similar findings were found across different doses of input virus, following both the percentage of E2^+^ cells and extracellular viral load (Fig 1C and 1D). To confirm the impact of mTOR activity on CHIKV infection, we tested PP242, an ATP-competitive mTOR inhibitor that specifically targets the active site of mTOR kinase, and again observed enhanced CHIKV infection (S1A and S1B Fig). These results suggest that mTOR activity restricts CHIKV infection, and highlighted an important paradox. While viral mRNA employs cap-dependent translation to replicate [7], CHIKV infection is actually increased when mTOR is inhibited.

To ascertain the mTOR molecular complex responsible for these findings, we selectively inhibited mTORC1 and mTORC2 by silencing *raptor* or *rictor*, respectively (Fig 1E and 1F). Inhibition of gene expression of *raptor*, but not *rictor*, recapitulated the enhanced CHIKV infection (Fig 1G). Confirming these results, we observed enhanced E2^+^ cells, increased expression of CHIKV proteins and higher extracellular viral load when cells were treated with the mTORC1 inhibitors Rapamycin or TORISEL (Fig 1H–1K). Importantly, a similar outcome was observed in cells with reduced expression of *rictor* (S2 Fig), demonstrating that Rapalog affected CHIKV infection independently of mTORC2. Together, these data indicate that inhibition of mTORC1, but not mTORC2, enhances CHIKV infection *in vitro*.

Using a complementary approach, we next evaluated the impact of mTORC1 on CHIKV infection by enhancing mTORC1 activity. This was achieved by inhibiting the expression of *tuberous sclerosis 2* (TSC2) gene, a physiologic inhibitor of mTORC1 (S3A Fig). When cells were treated with *tsc2* siRNA, the percentage of E2^+^ cells was decreased, as compared to cells treated with si-control (S3B Fig). This was validated using real time microscopy, which indicated that reduced gene expression of *tsc2* significantly restricted CHIKV propagation (S3C Fig). As an additional control for potential off-target effects, we demonstrated that Rapalog exposure overcame the inhibitory effect observed in *tsc2* si-RNA treated cells (S3D Fig). These results provide evidence for mTORC1 as an antiviral mechanism in the context of CHIKV infection. Following these results, we were interested to examine the possible interplay between mTORC1 and two effector pathways that were previously shown to impact CHIKV replication: type I IFN production; and autophagy initiation.

### Effect of mTORC1 on CHIKV infection is independent of type I interferon production

CHIKV induces rapid production of type I interferon (IFN) [16]. As several groups have reported that mTOR regulate interferon expression and mRNA translation of IFN-stimulated genes (ISGs) [17,18], we tested if mTORC1 inhibition increased CHIKV infection in a type I IFN dependent manner. We investigated the impact of Rapalog treatment in MEF deficient for both interferon regulatory factor (IRF) 3 and IRF7, two proteins essential for the production of type I IFN in CHIKV infected MEFs [16]. Remarkably, we observed that even in absence of *irf3* and *irf7* (*irf3* ^*-/-*^ */ irf7* ^*-/-*^), Rapamycin or TORISEL treatment resulted in increased CHIKV infection (S4A Fig). Similar results were obtained using interferon-α/β receptor (IFNAR) deficient MEF (S4A Fig).

We also analyzed the impact of mTORC1 inhibition on NF-κB-mediated inflammation. Indeed, Rapalog treatment did not perturb the expression levels of IκBα or the activation state of NF-κB (p-p65) (S4B Fig). Moreover, NF-κB-mediated cytokines were secreted at a similar level in untreated or TORISEL-treated cells, supporting that mTORC1 inhibition did not influence NF-κB pathways **(** S4C Fig). To assess other potential host response pathways, we queried whether the TORISEL-mediated enhancement of CHIKV infection is dependent on host cell transcription. Infected cells were exposed to TORISEL in the presence or absence of actinomycin D (ActD). Remarkably, the fold induction of CHIKV-GFP expression stimulated by TORISEL was unaffected by inhibition of transcription (S4D and S4E Fig). Based on these data, we excluded type I IFN or other host induced immune responses as the mechanism by which mTORC1 regulates CHIKV infection.

### Effect of mTORC1 on CHIKV infection is independent of autophagy

mTORC1 is a well-known inhibitor of macroautophagy (referred to as autophagy), a bulk degradation pathway that controls clearance and recycling of intercellular constituents for the maintenance of cellular survival [19]. Previously, we and others showed that CHIKV-induced autophagy serves as a mechanism of host defense by favoring cell survival and thereby restricting viral spread [9,20]. To test the hypothesis that mTORC1 enhances CHIKV infection in an autophagy dependent manner, we assessed viral infection in MEFs deficient for key autophagy genes *Atg5* (autophagy-related gene 5) or *Atg7*. Interestingly, autophagy deficient cells still exhibited increased infection following Rapamycin or TORISEL treatment (S5A–S5D Fig). Expression of *Atg5* and *Atg7* genes were also silenced in human foreskin fibroblastic cell line (HFF), with results indicating that the mTOR phenotype is indeed independent of autophagy and that the effect is not species specific (S5E–S5G Fig). Of note, while segregated from the mTOR effect, we did confirm that inhibition of autophagy genes significantly increased CHIKV infection, as previously reported [9,21].

### Rapalog treatment results in enhanced CHIKV disease

**Fig 2:**
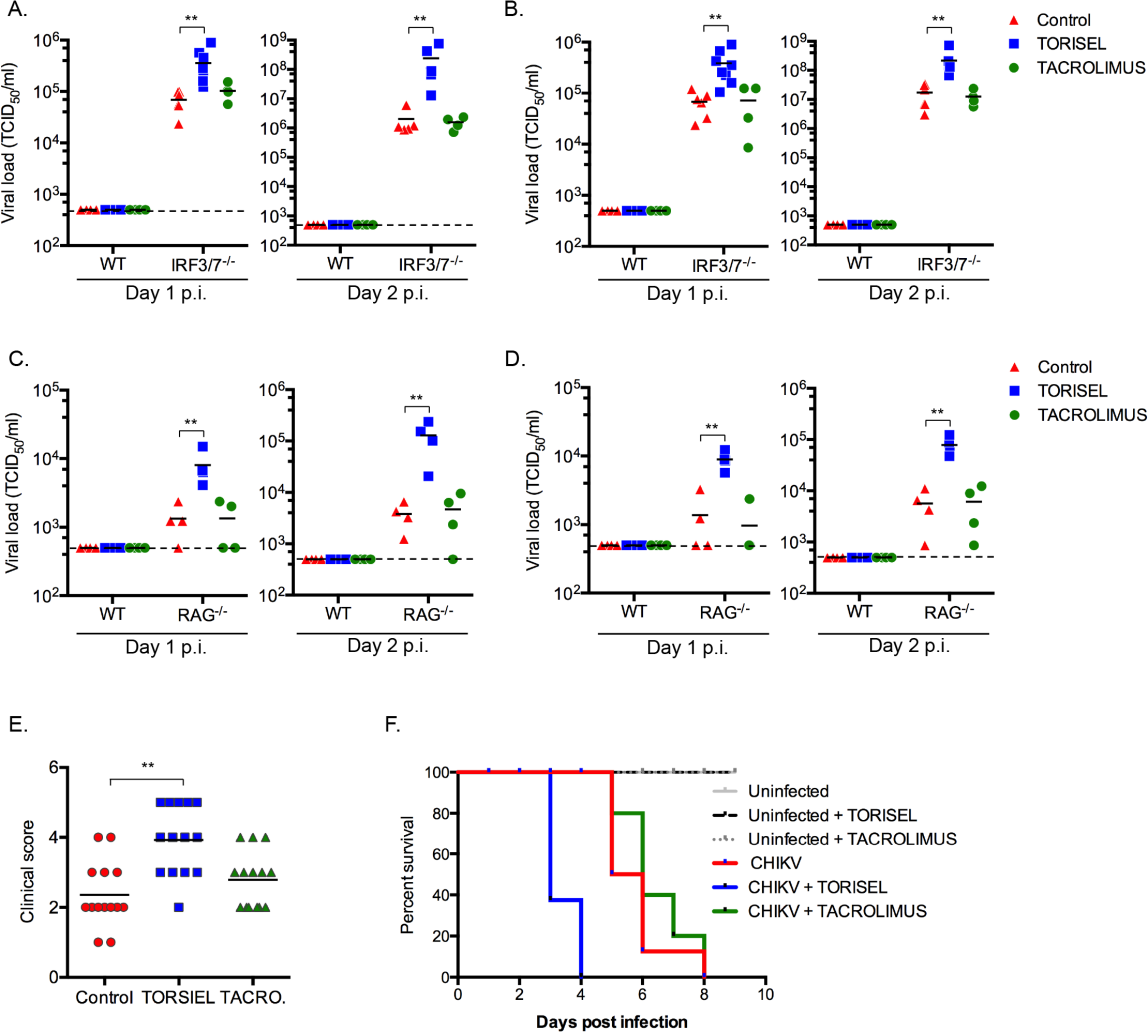
TORISEL favors CHIKV replication in infected tissues and enhances sensitivity of mice to infection. (**A-D**) Wild-type (WT) or IRF3^-/-^/7^-/-^ mice were treated with intra-peritoneal injection of 100 μL of solution containing TORISEL (10 mg/kg), tacrolimus (1 mg/kg) or PBS for 8 days followed by infection with 1 × 10^5^ PFU CHIKV, delivered subcutaneously. (**A, C**) Skin and (**B, D**) muscle were collected at indicated time points p.i., homogenized, and tested for viral titer. Median values for control (red), TORISEL (bueu) and tacrolimus (green) treatment are depicted. (**E, F**) IRF3^-/-^/7^-/-^ mice were treated with intra-peritoneal injection of 100 μL of solution containing TORISEL (10 mg/kg), tacrolimus (1 mg/kg) or PBS. (**E**) 2 days p.i., clinical score was assessed using a scale developed by K. Racke (score (1) = no change; score (2) = mouse do not grasp the cage with toes but with the ankle; score (3) = mouse is unable to return and land on its feet when flipped over; score (4) = hind limbs drag behind during walking or are not used by the animal for movement; score (5) = premoribund status). Individual mice are shown; median value is indicated by bar ±SEM. (**F**) Mice were monitored for lethal CHIKV for 10 d with data displayed as Kaplan-Meier curves (*n* = 14 for control mice, for TORISEL-treated mice and for Tacrolimus-treated mice). Student’s test **, P < 0.05.

To study the impact of rapalog treatment on *in vivo* CHIKV pathogenesis we used mice lacking the ability to produce type I IFNs, as wild type (WT) adult animals are resistant to severe forms of infection [22]. With the knowledge that mTOR acts independently of type I IFN expression, it was possible to employ *irf3* ^*-/-*^ *x irf7* ^*-/-*^ double deficient mice as a means to evaluate the impact of mTOR inhibition. This mouse strain is highly sensitive to CHIKV infection, with adult mice succumbing by day 6 post-infection [21]. After 8 days of intra-peritoneal injection of TORISEL (10mg/kg, injected every 2 days), *irf3* ^*-/-*^ */ irf7* ^*-/-*^mice were infected by CHIKV and tissues were analyzed at day 1 and 2 post-infection. We first validated that TORISEL inhibited mTOR activity in the skin and muscle of treated mice (S6 Fig). Consistent with our *in vitro* data, mice pre-treated with TORISEL had higher viral titers in both the skin (injection site) and muscle as compared to control mice, indicating that mTOR inhibition affects *in vivo* viral infection (Fig 2A and 2B). As one experimental caveat concerns the immunosuppressive effects of TORISEL, we nonetheless examined T and B cell inhibition as a confounding factor in our experiments. Notably, treatment with tacrolimus, a related FKBP-interacting immunosuppressive drug, did not affect viral titers in infected tissue (Fig 2A and 2B). These observations were further confirmed using mice deficient for *rag2* (*rag2* ^*-/-*^), and therefore lacking mature lymphocytes (Fig 2C and 2D). Finally, we observed a worsening of disease in TORSIEL treated animals, and a more rapid time to death of infected mice (Fig 2E and 2F). These experiments highlight the marked outcome of mTORC1 inhibition and enhanced viral replication, which lead to exacerbation of chikungnuya disease.

### mTORC1 specifically influences the CHIKV replication phase

**Fig 3:**
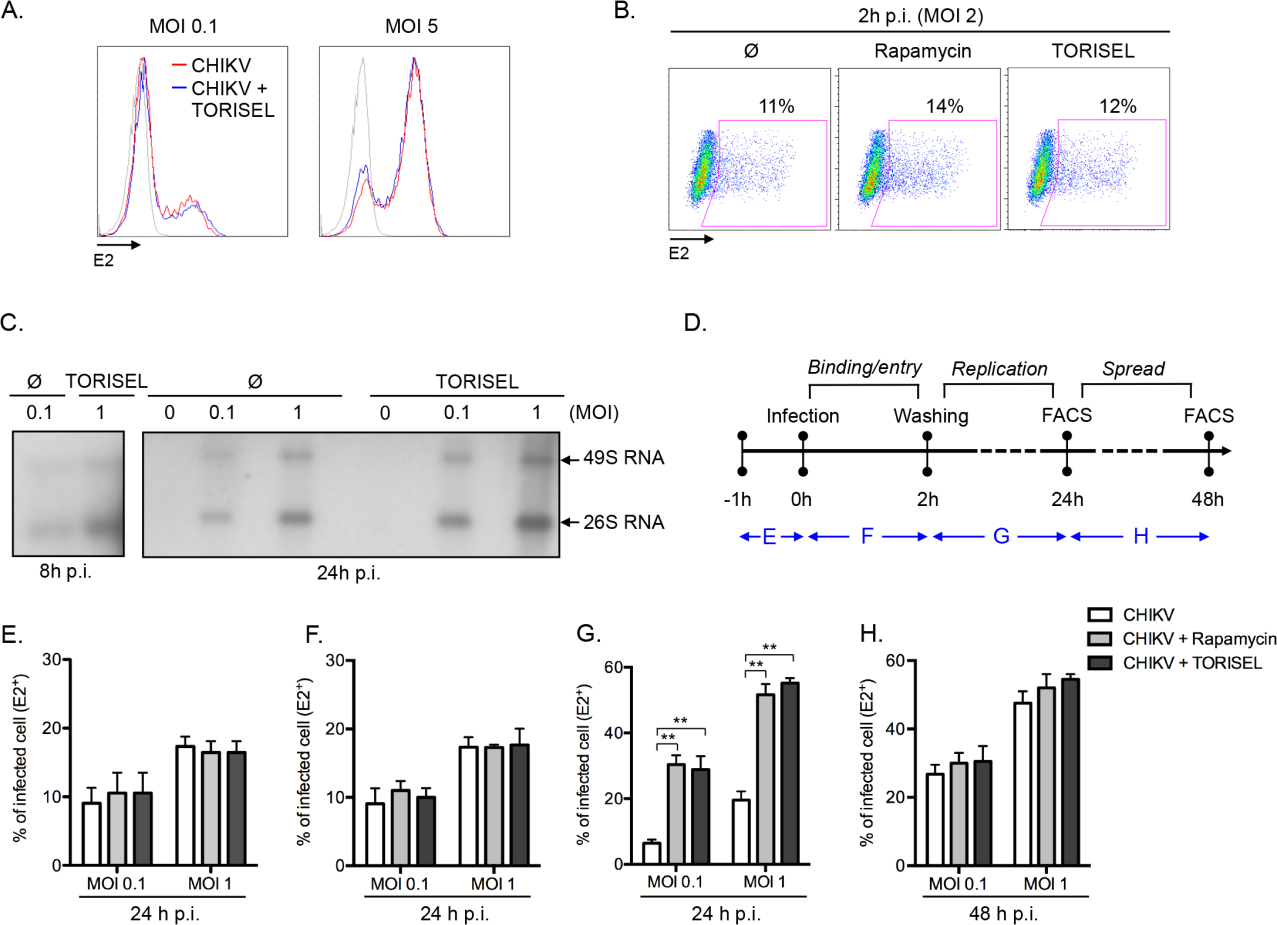
Rapalog treatment enhances viral replication. **(A)** MEFs were pretreated with TORISEL (0.1 mg/ml) then infected with CHIKV for 1h at 4°C. Extracellular E2 staining was analyzed by flow cytometry. Representative E2 staining is shown by histogram. Similar results were obtained in four independent experiments. **(B)** MEFs were pretreated with Rapamycin (100 nM) or TORISEL (0.1 mg/ml) for 24h and then infected with CHIKV (MOI = 2) for 2h. Intracellular E2 staining was analyzed with similar results obtained in four independent experiments. **(C)** MEFs were infected with CHIKV using indicated MOI in presence or absence of TORISEL (0.1 mg/ml). Northern blots were performed using specific radioactive probes that recognize 49S genomic or 26S subgenomic mRNA of CHIKV. Similar results were observed in three independent experiments. **(D-H)** MEFs were infected with CHIKV for 24h with indicated MOI in presence of Rapamycin (100 nM) or TORISEL (0.1 mg/ml). Blue bars indicate the period of Rapalog exposure. At the end of Rapalog treatment, cells were washed and maintained in control media or analyzed using flow cytometer depending of the time point **(D)**. **(E-H)** E2 positive cells were represented in graphs corresponding to the time points of Rapalog exposure: 1h pretreatment, prior to infection (E); treated 2h during CHIKV infection phase (F); treated post-infection for 22h (G); or treated 24h p.i. for an additional 24h (G). Bars indicate mean values ±SEM from three independent experiments. Student’s test **, P < 0.05. NS, non significant.

Having ruled out the two expected mechanisms (type I IFN and autophagy) by which mTORC1 could regulate viral infection, we addressed the mechanism of action using an unbiased approach, interrogating the effect of mTOR inhibition on the binding, entry, replication and/or spread of CHIKV. A direct analysis of viral binding (at 4°C) using a FACS-based assay showed that Rapalog treatment did not affect CHIKV binding (Fig 3A). Similarly, after a short period of infection (2h p.i. at 37°C), no difference was observed in intracellular staining of E2 (*i*.*e*., quantification of E2 present within the input virus) when comparing Rapalog-treated and untreated cells (Fig 3B). However, quantification of CHIKV genome during the first 24h of infection, showed a higher amount of both positive strand 49S genomic and subgenomic 26S viral mRNA in TORISEL-treated cells as compare to untreated cells (Fig 3C). Notably, a kinetic assessment of the timing for which Rapalog treatment influences CHIKV infection supports the conclusion that mTORC1 has its maximum impact on the CHIKV replication step (Fig 3D–3H). Indeed, binding and entry occur during the first hours of infection, and transient Rapalog exposure 1h pre-infection (Fig 3E), or two hours post-infection (Fig 3F), did not impact the eventual rate of infection. Only mTORC1 inhibition of during the first 24h of infection resulted in increased proportion of E2 positive cells (Fig 3G), yet treatment after the initial 24h of infection showed no additional impact (Fig 3H). These results suggest that mTORC1 activity specifically target the viral replication phase.

### Inhibition of mTORC1 improves the translation of both nonstructural and structural proteins

**Fig 4:**
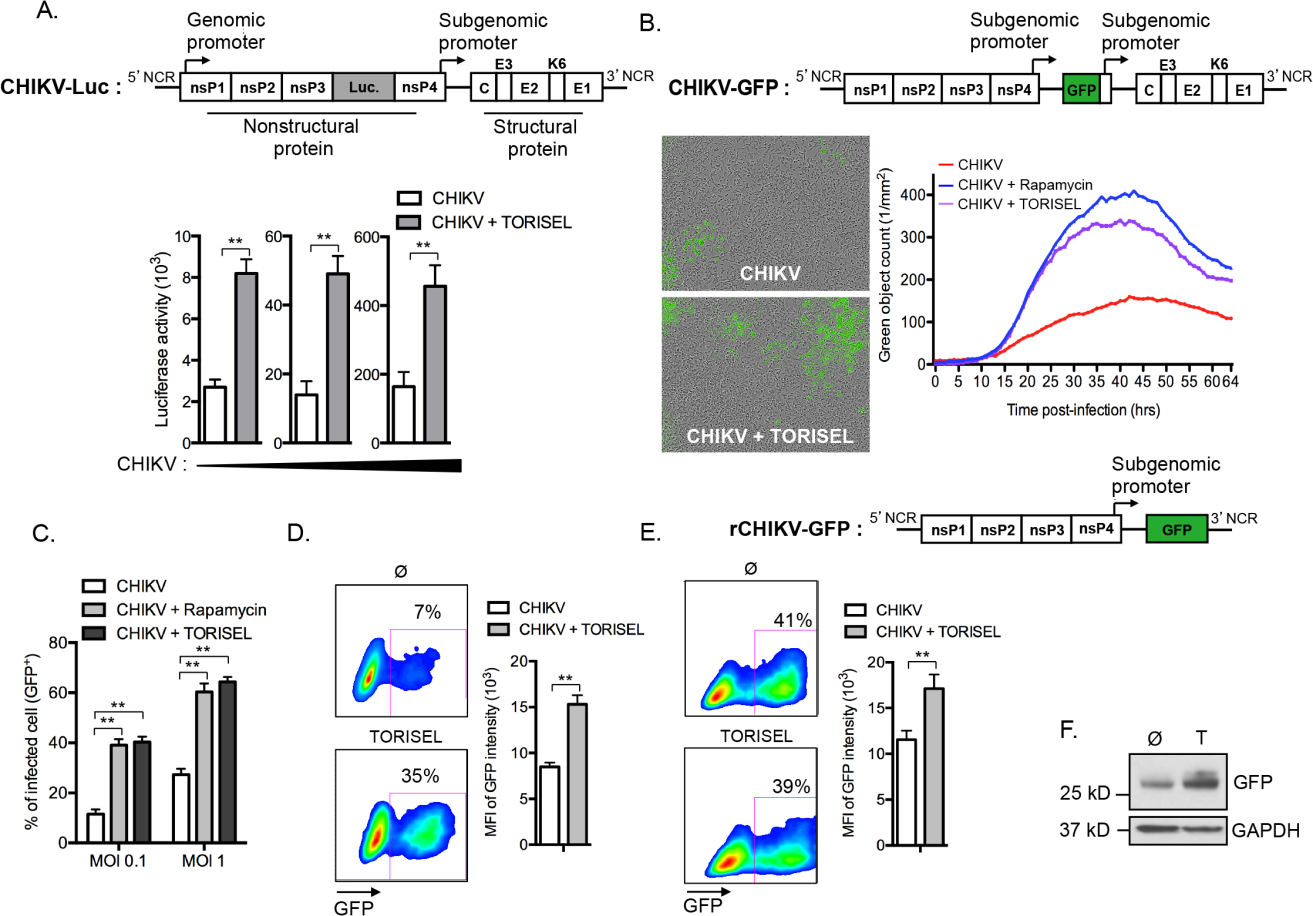
mTORC1 inhibitors enhance translation of both structural and non-structural CHIKV proteins. **(A)** MEFs were infected with increasing doses of CHIKV-luciferase reporter construct in the presence of TORISEL. A schematic of the CHIKV-luciferase construct is depicted. Luciferase activity was measured 4h p.i. using luciferin and read-out on a luminometer. Bars indicate mean values ±SEM from three independent experiments. **(B)** A schematic of the CHIKV-GFP construct is illustrated. MEFs were infected with CHIK-GFP (MOI = 1) in the presence of Rapamycin (100 nM) or TORISEL (0.1 mg/ml). GFP positive cells were quantified using real time imaging. Images show GFP staining (used to define green object count) and curves plot the kinetics of infection. Similar results were obtained in seven independent experiments. **(C)** MEFs were infected with CHIKV-GFP for 24h at indicated MOI in presence of Rapamycin or TORISEL, and the percentage of GFP positive cells were determined. Similar results were observed in five independent experiments. **(D)** WT MEFs were infected with CHIKV-GFP (MOI = 1) for 24h in presence of TORISEL. Infected cells were gated according to GFP intensity and the MFI was plotted for infected cell populations. Bar indicate mean values ±SEM from three independent experiments. **(E)** A schematic of the single cycle rCHIKV-GFP construct is shown. MEFs were transfected with rCHIKV-GFP and after 24h TORISEL was added to the cultures. GFP positive cells were gated by flow cytometry and the MFI was plotted. Bars indicate mean values ±SEM from three independent experiments. **(F)** Cell lysates were prepared from rCHIKV-GFP transfected MEFs and Western blot was performed using anti-GFP and anti-GAPD antibodies. Similar results were observed in two independent experiments. Ø, control; T, TORISEL. Student’s test **, P < 0.05.

Following the evidence for Rapalog exposure acting to increase viral replication, we investigated whether enhanced translation of nonstructural proteins (nsP) accounts for the higher viral mRNA replication. To monitor nonstructural protein translation, we used a reporter CHIKV encoding luciferase under the control of the genomic promoter (CHIKV-Luc, construct illustrated in Fig 4A). Strikingly, TORISEL exposure resulted in a 3–4-fold increase in luciferase activity for all viral doses tested (Fig 4A). Notably, these results were obtained 4h post-infection, a time point prior to the completion of CHIKV replication; thereby ensuring Luciferase activity is an accurate measure of nonstructural protein translation. These data support Rapalog treatment acting in a cell-intrinsic manner to enhance the translation of viral nonstructural proteins.

As the genomic ORF (encoding for nonstructural proteins) and subgenomic ORF (encoding for structural proteins) of CHIKV utilize different promoters, we next assessed if the inhibition of mTORC1 could also improve the translation of structural proteins. To accomplish this, we utilized a recombinant CHIKV expressing GFP under the control of the subgenomic promoter (CHIKV-GFP 5’ construct illustrated in Fig 4B), and performed hourly monitoring of infection using real time microscopy. Results confirmed that Rapalog exposure increases infection (Fig 4B and S1 and S2 Movies). Similar results were obtained by examining the percentage of GFP positive cells using cytometric analysis, showing that Rapalog treatment similarly affects the recombinant CHIKV-GFP 5’ and wild type CHIKV (Fig 4C as compared to Fig 1). Importantly, evaluation of GFP expression in infected MEF (GFP^+^ cells) indicated that TORISEL-treatment increased both the percentage of GFP expressing cells (7 to 35%, p-value = 0.025), and the per cell expression of GFP (8500 to 15200 MFI, p-value = 0.014, Fig 4D). We confirmed this result by using a truncated form of CHIKV, lacking the subgenomic region (rCHIKV-GFP construct illustrated in Fig 4E). This defective GFP reporter virus permitted us to monitor the translation of structural proteins at later time points. At 24h post-transfection, cells were treated with TORISEL and GFP expression was analyzed after an additional 24h (Fig 4E). While the efficiency of transfection was similar (41% vs. 39%), TORISEL-treated cells exhibited a higher expression of GFP protein as compared to untreated cells (11750 to 16450 MFI, p-value = 0,026, Fig 4E and 4F). Therefore, Rapalog treatment acts to enhance translation of both nonstructural and structural CHIKV proteins, and mediates its activity in a cell autonomous manner.

### Alphavirus protein translation is enhanced despite a global reduction in cellular protein translation

**Fig 5:**
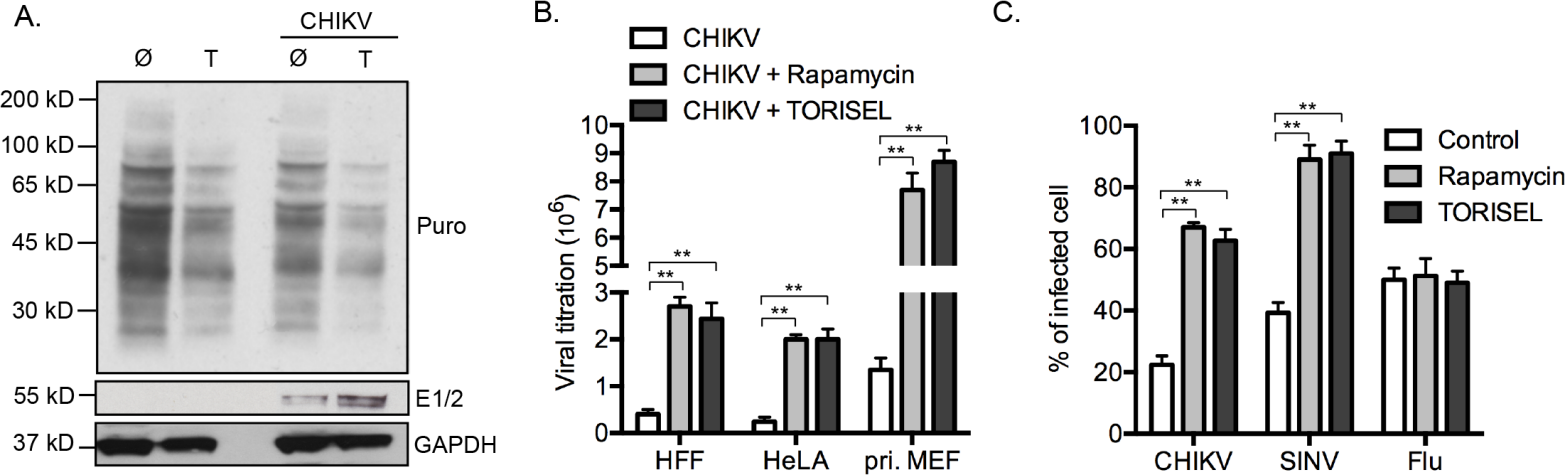
Enhanced protein translation during mTORC1 inhibition is specific for viral proteins. **(A)** MEFs were infected with CHIKV (MOI = 1) in the presence of TORISEL for 24h (0.1 mg/ml) and labeled with puromycin (Puro) as detailed in the Methods section. Western blot was performed using anti-puromycin (clone 12D10), anti-E1/2 and anti-GAPD antibodies. Similar results were observed in three independent experiments. **(B)** HFF, HeLA or primary MEFs were infected with CHIKV (MOI = 0.1) in the presence of Rapamycin (100 nM) or TORISEL (0.1 mg/ml). Extracellular viral titers were determined 24h p.i. and results expressed as TCID_50_/ml. Bars indicate mean values ±SEM from four independent experiments. **(C)** MEFs were infected with CHIKV (MOI = 1), Sindbis expressing GFP (SINV; MOI = 0.1) or influenza A (Flu, 250 HAU10^6^) for 24 h. The staining of E2, GFP and M2 proteins were analyzed using flow cytometry to quantify infection. Bars indicate mean values ±SEM from three independent experiments. Ø, control; T, TORISEL. Student’s test **, P < 0.05.

Regarding the central role for mTORC1 on the activation of cap-dependent translation, our results showed a paradoxical effect of Rapalog on CHIKV proteins translation. To determine if this effect is selective to viral proteins, we investigated the global state of host protein translation (Fig 5A). Using the SUnSET method [23], we showed that TORISEL exposure decreased the global state of host protein translation, in both uninfected and infected cells. Confirming our findings, we show that in the same experiment, TORISEL treated cells expressed higher amount of CHIKV E1 and E2 (Fig 5A). These results suggest that CHIKV translation, despite it being cap-dependent, has evolved a mechanism to bypass mTORC1 inhibition.

Our results were extended to both human cell lines as well as primary human and mouse fibroblasts. Specially, human foreskin fibroblasts (HFF), human epithelial cells line (HeLa) and primary mouse fibroblast (pri. MEF) were infected by CHIKV, which all showed a similar increase in viral load when mTORC1 was inhibited (Fig 5B). Interestingly, the enhancement of virus titer was more pronounced in primary MEF as compared to cell lines (9–10-fold increase as compared to 3–4-fold increase), indicating a role for mTORC1 in physiologic conditions.

We next investigated if a similar effect of Rapalog treatment could be observed with other viral infection. We chose to study two different viruses: sindbis (SINV), a second member of alphavirus family with a replication cycle similar to CHIKV; and influenza A (Flu, strain A/Puerto Rico/8/1934 H1N1), a member of orthomyxoviridae family. Interestingly, while Rapalog exposure enhanced CHIKV and SINV infection, no effect was observed for Flu (Fig 5C). These results suggest that different viruses have established unique strategies for modulating mTORC1 activity and/or overcoming translational stop mediated by mTOR inhibition.

### CHIKV protein translation requires eIF4E activity

Several classes of proteins use a cap-independent translation mechanism which includes the expression of an internal ribosome entry site (IRES) or IRES-like structures [24]. Indeed, IRES are often used by viruses as a means to ensure that viral protein translation is active during cellular stress or other conditions leading to mTORC1 inhibition. For both the structural and nonstructural polyprotein ORF of CHIKV, no IRES or IRES-like structures have been identified identified, suggesting that CHIKV proteins are translated *via* a cap-dependent mechanism [5]. To test this prediction, we investigated infection efficiency in cells silenced for *eIF4E*, a protein essential for the initiation of capped mRNA [25]. MEF cells were pretreated with siRNA that targeted *eif4e* mRNA and infection efficiency was analyzed by flow cytometry and real time imaging (S7A and S7B Fig). Reduced eIF4E expression decreased the amount of CHIKV infected cells. Similarly, inhibition of the interaction between eIF4E and eIF4G, using the inhibitor 4EGI-1, markedly limited the translation of both structural and nonstructural CHIKV protein (S7C and S7D Fig). These results demonstrated a key role for eIF4E protein in CHIKV infection and supported the prior assumption that CHIKV proteins require a cap-dependent translation mechanisms to be processed.

### CHIKV protein expression bypasses mTORC1 inhibition *via* MnK / eIF4E mediated translation

**Fig 6:**
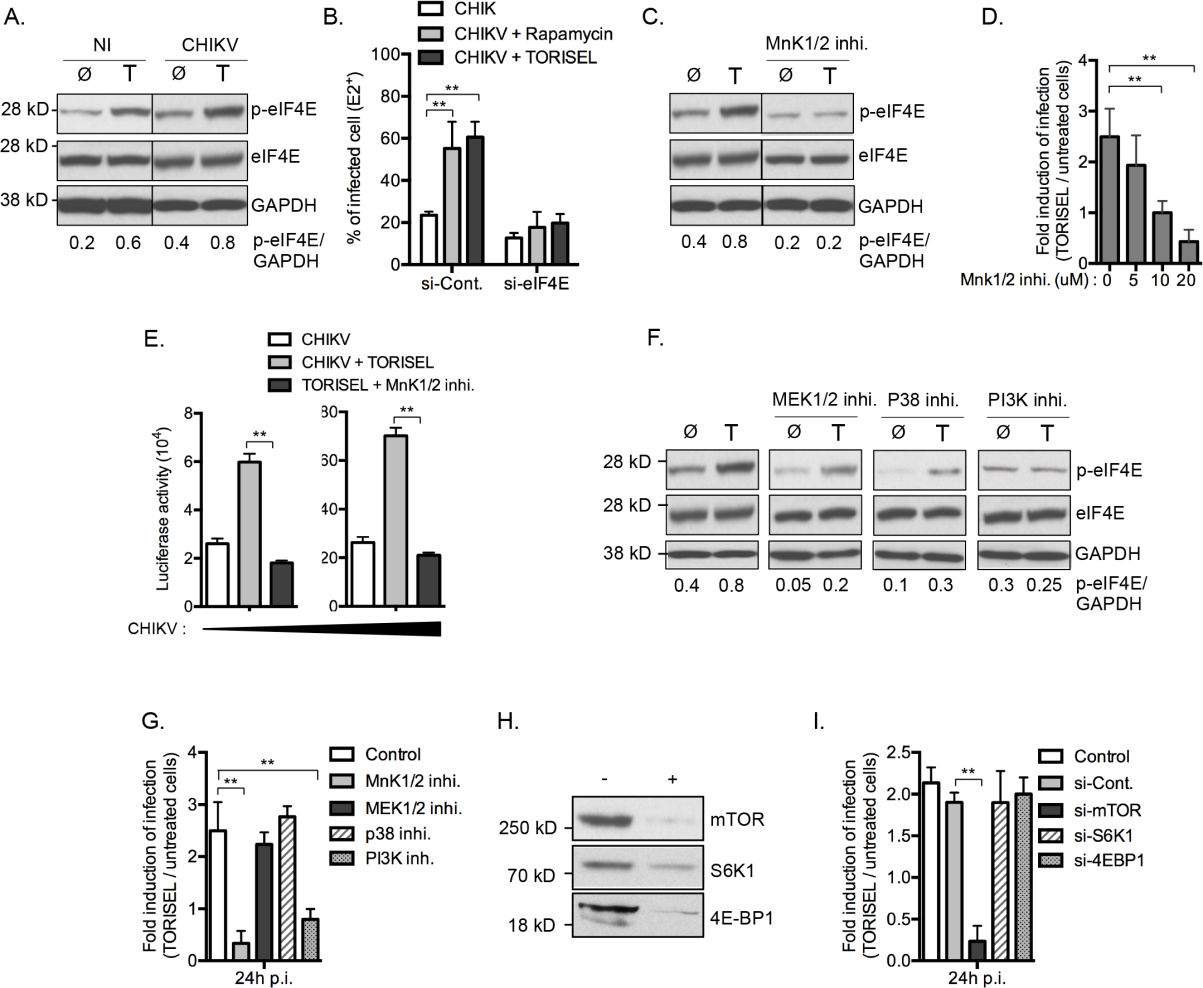
Inhibition of mTORC1 favors viral protein translation by activation of an MnK/p-eIF4E pathway. **(A)** MEFs were infected with CHIKV (MOI = 5) in the presence of TORISEL (0.1 mg/ml) for 24h and eIF4E phosphorylation was assessed by Western blot. Band intensity of the p-eIF4E/GAPDH ratio calculated with numbers shown below the respective conditions. Similar results were observed in three independent experiments. **(B)** MEFs were pretreated with *eif4e* siRNA followed by CHIKV infection (MOI = 1) in presence of Rapamycin (10 nM) or TORISEL (0.1 mg/ml). Percentage of E2 positive cells was measured 24 p.i.. Bars indicate mean values ±SEM from three independent experiments. **(C)** MEFs were infected with CHIKV (MOI = 5) in presence of TORISEL (T) and/or an MnK1/2 inhibitor (CGP57380, 20 μM) for 24h and Western blot analysis was performed to detect phosphorylation of eIF4E at serine 209 (p-eIF4E (S209)). eIF4E and GAPDH were measured to control protein expression and loading. Band intensity of the p-eIF4E / GAPDH ratio is reported. **(D)** MEFs were infected with CHIKV-GFP (MOI = 1) in the presence of TORISEL and/or indicated dose of an MnK1/2 inhibitor (CGP57380). GFP positive cells were analyzed 24 p.i. using real time imaging. Results represent the fold induction of GFP positive cells observed in TORISEL treated cells, as compared to untreated cells. Bars indicate mean values ±SEM from three independent experiments. **(E)** MEFs were infected with increasing doses of CHIKV-Luc in presence of TORISEL ± MNKs inhibitor II (MNK1/2 inhi. – 5 μM) and luciferase activity was measured at 4h p.i. Bars indicate mean values ±SEM from three independent experiments. **(F)** MEFs were infected with CHIKV (MOI = 5) for 24h in presence of TORISEL ± MEKs inhibitor (PD0325901, 1 μg/ml), an p38 MAPK inhibitor (SB203580, 1 μM) or an PI3K inhibitor (LY294002, 25 μM) and eIF4E phosphorylation was followed by Western blot. p-eIF4E / GAPDH band intensity is reported. Similar results were observed in three independent experiments. **(G)** MEFs were infected with CHIKV (MOI = 1) for 24h using the inhibitors described in (**E**). Results represent the fold induction of E2 positive cells observed in TORISEL treated cells, as compared to untreated cells. Bar indicate mean values ±SEM from three independent experiments. **(H, I)** MEFs were pretreated with siRNA specific for *mtor*, *s6k1* or *4e-bp1* followed by CHIKV infection (MOI = 1) in presence of TORISEL. Western blot was performed 24h p.i. using anti-mTOR, anti-S6K1 and anti-4E-BP1 antibodies **(H)**. Additionally, the fold induction of E2 positive cells is shown. Bars indicate mean values ±SEM from three independent experiments **(I)**. Ø means control; T means TORISEL. +, indicates respective siRNA knock-down;-, indicates si-control. Student’s test **, P < 0.05.

A Rapalog resistant mechanism is present in some tumor cells to maintain translation of capped mRNA when mTORC1 is inhibited [26,27]. This bypass mechanism requires phosphorylation of eIF4E at serine 209, increasing its binding affinity for the capped mRNA, and thereby favoring formation of translation initiation complexes [28]. To investigate the role of mTORC1 activity on eIF4E phosphorylation in our model, we first analyzed the amount of p-eIF4E in TORISEL-treated MEF (Fig 6A). As shown, cells treated with TORISEL expressed higher amount of p-eIF4E as compare to untreated cells, demonstrating that Rapalog treatment leads to increased eIF4E activation (Fig 6A). Importantly, similar results were observed at 24h post-infection, demonstrating that Rapalog exposure enhances the phosphorylation of eIF4E in uninfected and CHIKV infected cells (Fig 6A). The activation of eIF4E was directly linked to Rapalog-induced CHIKV infection by showing that si-*eif4e* transfected cells were resistant to Rapalog treatment (Fig 6B). These results demonstrated eIF4E activity is critical for increased CHIKV replication when mTORC1 is inhibited. Of note, despite TORISEL treatment leading to an increase in p-eIF4E in uninfected cells, the global amount of host protein translation was diminished (Fig 4A), suggesting that eIF4E activity is being preferentially co-opted by CHIKV.

MnK1/2 are the major kinases mediating phosphorylation of eIF4E [29]. We therefore asked whether TORISEL-induced phosphorylation of eIF4E was dependent on MnK proteins. This was performed using as an inhibitor for both MnK1 and MnK2 (CGP57380), which prevented the increase of p-eIF4E observed after TORSIEL treatment (Fig 6C). These results are consistent with prior reports of Rapalog treatment enhancing eIF4E phosphorylation via the activation of MnKs [28,30–32]. Importantly, using the recombinants CHIKV-GFP 5’ or CHIKV-luciferase, we demonstrated that infection and/or translation of CHIKV proteins were not influenced by TORISEL exposure in cells pretreated with MnKs inhibitor (CGP57380 or MnK1/2 inhibitor II) (Fig 6D and 6E). These findings indicate that mTORC1 inhibition favors CHIKV protein translation by an increased MnK-dependent phosphorylation of eIF4E.

We next studied activators of MnK1/2 in order to define the molecular pathway by which eIF4E is engaged. Strikingly, neither of two known kinases responsible for MnK activation, mitogen-activated protein kinase kinase (MAPKK also known as MEK) and p38 MAPK, were required for the enhanced phosphorylation of eIF4E or for the increase in CHIKV proteins translation (Fig 6F and 6G). Cross talk between the phosphatidylinositol-3 kinase (PI3K) and MnKs signaling has been previously reported in human cancer cells [32]. In the context of viral infection, we show that the PI3K inhibitor LY294002 blocked the Rapalog-induced activation of eIF4E and the enhancement of CHIKV (Fig 6F and 6G), suggesting that mTOR inhibition increases eIF4E phosphorylation and subsequently the CHIKV infection through a PI3K-dependent and MnK-mediated mechanism.

To investigate potential co-regulation of mTORC1 among the different effector pathways, we analyzed the role of S6K1 and 4E-BP1. Notably, silencing of these respective genes did not impact Rapalog-induced CHIKV infection (Fig 6H and 6I). Together, these data indicate that mTORC1 has a direct impact on PI3K and the MnK/p-eIF4E pathway.

### CHIKV-mediated inhibition of mTORC1 favors infection through a MnKs/p-eIF4E dependent pathway

**Fig 7:**
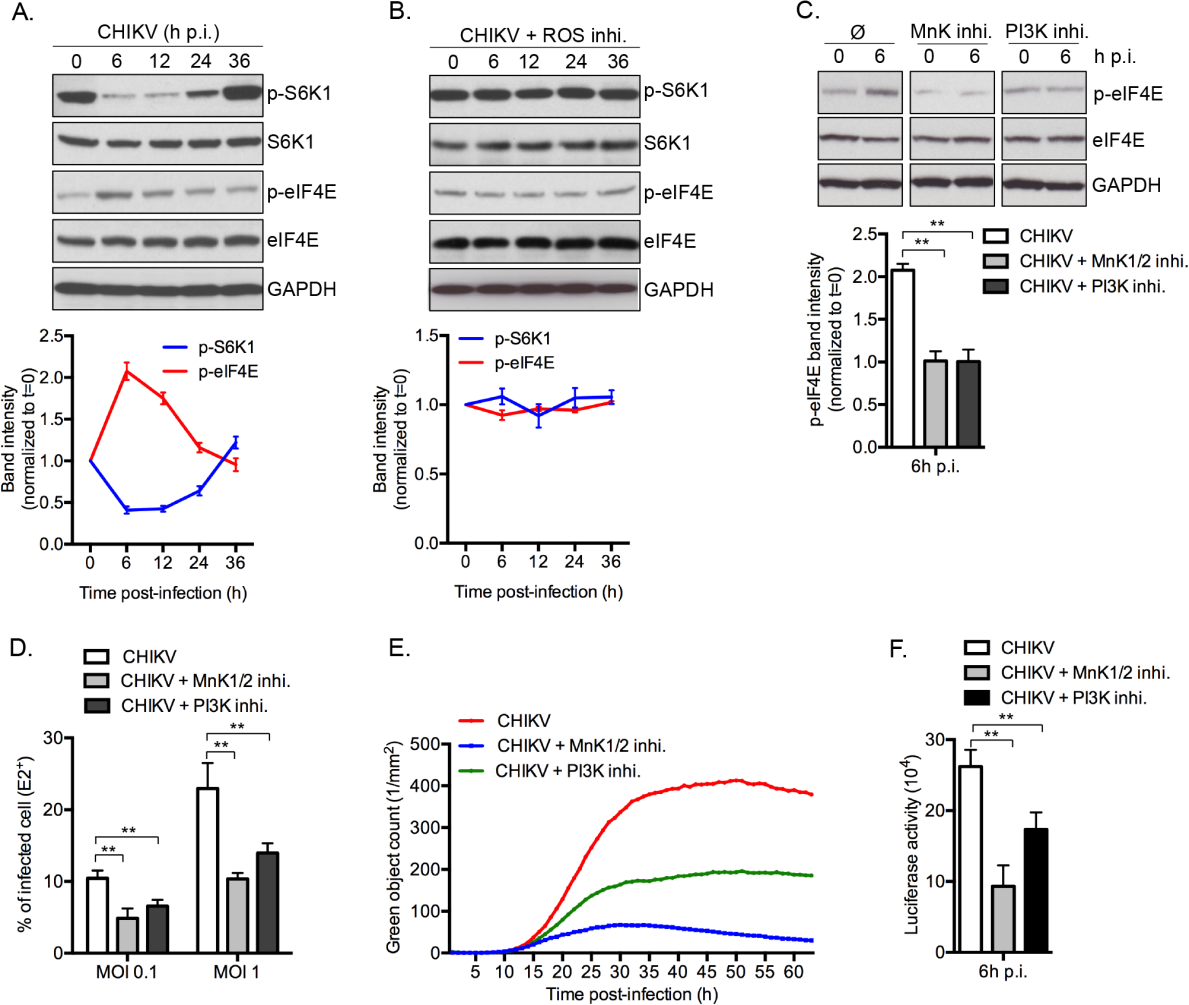
CHIKV-induced mTORC1 inhibition favors infection through the MnK/p-eIF4E pathway. **(A)** MEF were infected with CHIKV (MOI = 5) and phosphorylation of S6K1 and eIF4E was assessed by Western blot at indicated times post-infection. Band intensities for p-S6k1 and p-eIF4E were normalized to t = 0 and as a function of time (red line, p-eIF4E, blue line, p-S6K1). Bars indicate mean values ±SEM from three independent experiments. **(B)** MEF were infected with CHIKV (MOI = 5) in presence of ROS inhibitor (*N*-acetyl-*L*-cysteine) and phosphorylation of S6K1 and eIF4E was followed as in (A). **(C)** MEF were infected with CHIKV (MOI = 5) in presence of an MnK inhibitor (MnK1/2 inhi. – 5 μM) or PI3K inhibitor (LY294002, 25 μM) and phosphorylation of eIF4E was followed at 6h p.i.. Band intensity for p-eIF4E normalized to t = 0. Bars indicate mean values ±SEM from three independent experiments. **(D, E)** MEFs were infected with CHIKV at indicated MOI in the presence of MnK inhibitor (MnK1/2 inhi. – 5 μM) or PI3K inhibitor (LY294002, 25 μM), and the percentage of intracellular E2 staining was analyzed at 24h p.i. using an anti-E2 and monitored by FACS analysis. Bars indicate mean values ±SEM from four independent experiments (**D**). In parallel, MEFs were infected with CHIKV-GFP (MOI = 1) and GFP positive cells were followed using real time imaging. Similar results were obtained in five independent experiments (**E**). **(F)** MEFs were infected with CHIKV-Luc (MOI = 1) in presence of MnK inhibitor or PI3K inhibitor. Luciferase activity was determined at 6h p.i.. Bars indicate mean values ±SEM from three independent experiments. Ø, control. Student’s test **, P < 0.05.

To demonstrate the physiologic relevance of our discovery, we investigated the role of CHIKV-mediated inhibition of mTORC1 on infection. Interestingly, inhibition of mTORC1 correlated with an increase of eIF4E activity, with peak phosphorylation occurring at 6 h post-infection (Fig 7A). These data advances our previous report of a rapid and transient inhibition of mTORC1 during CHIKV infection, regulated by CHIKV-mediated ROS production and AMPK activation [9]. We also demonstrate that preventing CHIKV-mediated inhibition of mTOR, using ROS inhibitor (*N*-acetyl-*L*-cysteine), abrogated the enhanced p-eIF4E (Fig 7B). Moreover, inhibition of PI3K or MnKs, using respective inhibitors, abolished the CHIKV-mediated phosphorylation of eIF4E (Fig 7C). Together, these results show that CHIKV infection increases the phosphorylation of eIF4E through a PI3K and Mnk1/2 dependent.

**Fig 8:**
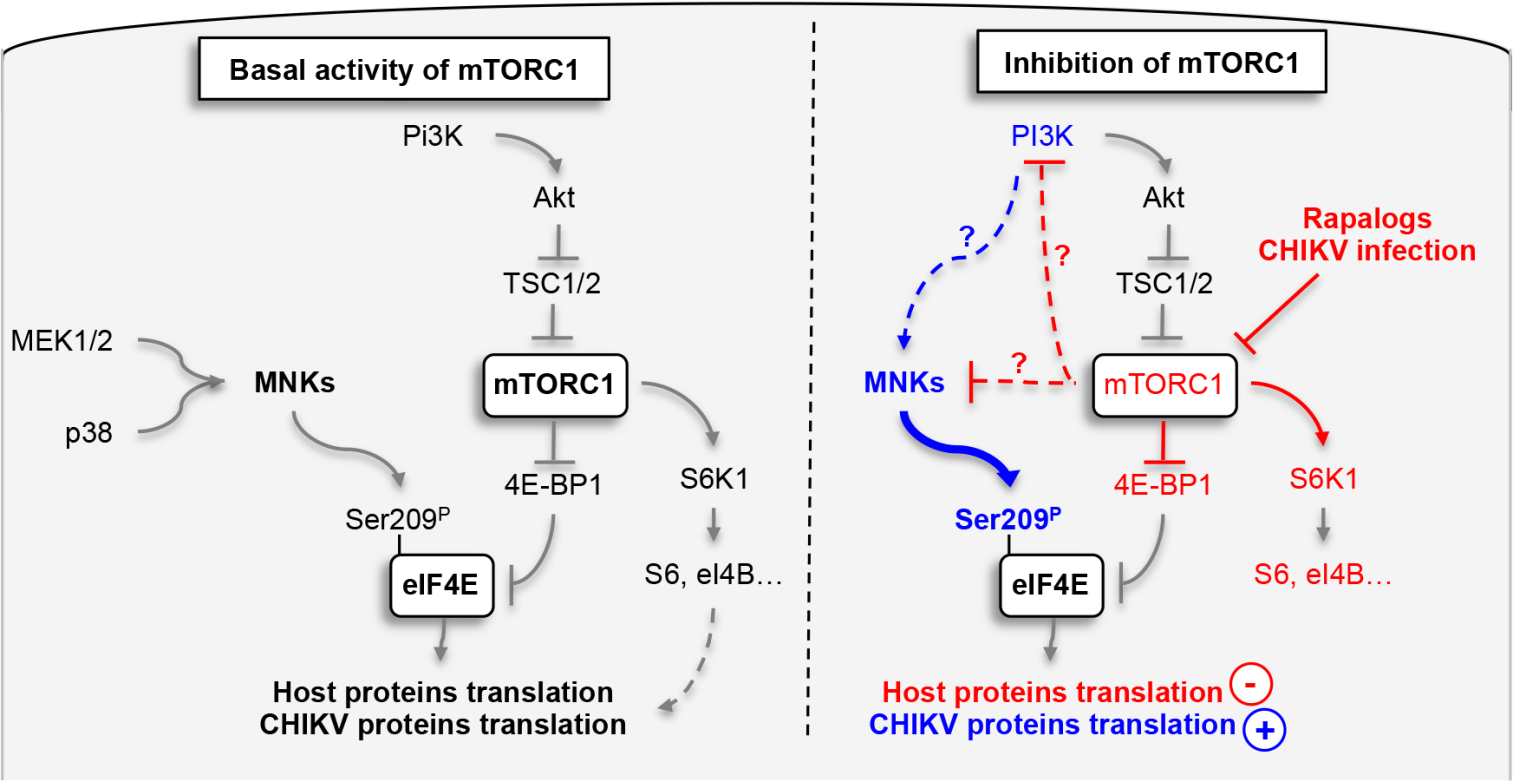
Schematic representation of mechanism by which CHIKV protein synthesis is increase by the inhibition of mTORC1. Grey lines indicate pathways known in the literature; blue and red lines highlight respectively activation and inhibition pathways characterized in this study. Indirect molecular connections are indicated by dotted lines.

Finally, to define the impact of phosphorylation of eIF4E on natural CHIKV infection, we tested the direct impact of MnK or PI3Ks inhibitors on viral protein expression and viral spread (Fig 7D–7F). Interestingly, inhibition of either Mnk or PI3K significantly decreased the percentage of E2 positive cells (Fig 7D), the translation of structural (Fig 7E) and nonstructural proteins (Fig 7F). These data support our conclusion that CHIKV-induced activation of eIF4E favors infection during natural infection, and also explains the enhancement of viral infection when mTORC1 is pharmacologically inhibited by Rapalog treatment (Fig 8).

## Discussion

Herein, we evaluated the role of the metabolic regulator mTOR during CHIKV infection. To our surprise, inhibition of mTORC1 enhanced viral protein translation *via* a mechanism that is independent of autophagy and type I IFN signaling. Being an important initiator of cap-dependent protein translation, the inhibitory effect of Rapalog treatment on viral has been well documented in instances where the virus possesses capped mRNA [14,33]. The results from our current study, however, identify an unexpected effect of mTORC1. During natural CHIKV infection, mTORC1 limits viral replication despite CHIKV requiring a host mRNA cap. This was further evident when cells were exposed to Rapalog, which resulted in a massive induction of CHIKV protein expression. Indeed, our study provides the first evidence for direct enhancement of viral protein translation during mTORC1 inhibition, thus revealing a new strategy developed by CHIKV to maintain viral protein expression in the context of cell stress.

### A new viral bypass for the host translational machinery

As a metabolic sensor, mTOR activity is perturbed during viral infection [12]. To ensure viral protein translation, viruses have evolved strategies to bypass cellular programs that limit the ribosomal machinery. Regulation by mTORC1 has been a major focus and prior work has illustrated the different mechanisms evolved in the context of the host / microbe détente. For example, HSV-1 enhances mTORC1 activity; whereas Poliovirus, HIV-1, Sindbis and CHIKV inhibit this complex [12]. For viruses inducing a stop in protein translation, they have means to ensure translation of their own mRNAs. Most strategies identified thus far involve the use of non-canonical translation mechanisms, including IRES, ribosome shunting or VPg [24]. These processes permit efficient viral replication in absence of the cap-dependent translation initiation protein eIF4E.

To demonstrate that CHIKV proteins use a classical cap-dependent process, we analyzed the dependence on the active form of eIF4E. Indeed, absence of eIF4E expression lost of eIF4E phosphorylation or abrogation of eIF4E/eIF4G complex formation led to a drastic inhibition in CHIKV infection (Figs 7 and S7). Thus CHIKV has evolved a newly discovered mechanism of bypassing mTORC1 inhibition. Notably, we show that a similar strategy seems to be employed by Sindbis (Fig 5C)–consistent with its replication requiring an active form of eIF4E [18]–but not by influenza A (Fig 5C), whose infection proceeds normally even when eIF4E is functionally impaired [34].

To achieve translation initiation, CHIKV engages an MnK-dependent hyper-phosphorylation of eIF4E. This mechanism has been reported in tumors, providing a means to support cap-dependent protein translation under conditions of cell stress [30,31]. In our model, however, hyper-phosphorylation of eIF4E does not restore translation of host proteins, which is shutdown after Rapalog exposure (Fig 5A). This defines a selective strategy by CHIKV to enhance its viral protein translation *via* p-eIF4E, overcoming mTORC1 inhibition of conventional cap-dependent pathways.

### Molecular connections between mTOR and MNKs pathways

The phosphorylation of eIF4E and the formation of the eIF4E/eIF4G complex are tightly regulated by signal transduction pathways that converge on MnK1/2 and mTOR [35]. While both pathways are known to positively impact translation initiation, interconnections between the two signaling mechanisms have remained unclear. Several investigations suggested that the MnKs are activated by Rapalog treatment, resulting in the maintenance of cap-dependent translation during inhibition of mTORC1 [30,31], however the molecular scaffolds are not known.

In the present study, we demonstrated that activation of MnK1/2, induced by Rapalog or CHIKV-mediated mTOR inhibition, results in upregulation of viral protein translation *via* the hyper-phosphorylation of eIF4E. We show that translation of CHIKV proteins are not only resistant to mTORC1 inhibition, but can be enhanced by this process *via* the activation of PI3K and the subsequent engagement of the MnK/eIF4E pathway. The effect of mTOR on CHIKV infection was independent of both S6K1 and 4E-BP1 signaling. These observations align with previous work published by Wang and colleagues, who reported that Rapamycin-induced activation of MnK is independent of S6K1 activity [32]. 4E-BP1 hypo-phosphorylation increases its binding to eIF4E and prevents the formation of eIF4F complex [10]. While we clearly show that Rapalog treatment induces hypophosphorylation of 4E-BP1, eIF4E-dependent translation is increased (Fig 1). Interestingly, in our model, PI3K inhibition was able to restrict CHIKV infection and limit the effect of Rapalog on virus replication. Since the classical downstream signals of mTORC1 (S6Ks and 4E-BPs) and upstream signal of MnKs (MEK and p38) are not involved in the Rapalog effect, molecular connections between mTOR, PI3K and the MnK pathway must be examined further.

### Role of mTORC1 activity during an acute CHIKV infection

In our previous report, we described a transient inhibition of mTORC1 pathway during the first hour of infection that led to an induction of autophagy that in turn limits CHIK-induced apoptosis [9]. In infected cells, mTORC1 is inhibited through the intrinsic production of ROS leading to the activation of both AMPK and the TSC1/TSC2 complex [36]. Herein, we confirmed the transient inhibition of mTORC1 during CHIKV infection and discovered a new function of mTOR as a direct regulator for viral protein translation. Indeed, preventing mTOR-dependent hyper-phosphorylation of eIF4E using MnK or PI3K inhibitors significantly decrease infection efficiency (Fig 7). Integrating our new findings, we suggest that CHIKV-mediated inhibition of mTORC1 benefits the virus by increasing the efficiency and timing of translation, *via* the activation of eIF4E and autophagy respectively.

Strikingly, while ROS inhibition prevents the transient inhibition mTORC1 (Fig 7B), we did not observe a significant difference for CHIKV infection in ROS inhibitor-treated *versus* untreated cells (S8 Fig). These results could be explained by the fact that ROS itself has an mTOR-independent antiviral effect on CHIKV infection, as illustrated by ROS inhibition leading to a marked increase of infection in siRNA *mtor* or Rapalog treated cells (S8 Fig). Having this new knowledge, we postulate that inhibition of mTORC1 balances the antiviral effect of CHIKV-mediated ROS production.

### mTOR participates in restricting viral replication

mTOR has been considered in other infectious models as important for host response. Pulendran and colleagues showed that the TLR9 ligand CpG-A triggered phosphorylation of mTOR and its downstream targets 4E-BPs and S6Ks [37]. Accordingly, TLR9-mediated production of type I IFN, tumor necrosis factor (TNF) and interleukin 6 (IL-6) was suppressed in human and mouse plasmacytoid dendritic cells (pDC) treated with Rapamycin [37]. Another strategy by which mTOR has been associated to immune antiviral response relates to its cross-talk with the autophagy pathway. Indeed, several viruses have developed strategies to use autophagososmes as a membrane support for viral replication and/or release, including HCV, poliovirus and HIV-1 [38,39]. In this context, inhibition of autophagy mediated by mTORC1 could limit viral infection.

Our results highlight a new antiviral mechanism for mTOR that is independent of IFN production and autophagy. We showed that inhibition of mTORC1 increased CHIKV infection in both *in vitro* and *in vivo* models. This process was based on direct regulation of viral protein translation and is independent of new transcriptional activity, as the effect of Rapalog treatment was unaffected by ActD exposure. Importantly, we also confirmed the impact of mTOR on CHIKV infection by increasing the activity of endogenous mTORC1. These results suggest that strategies aimed at enhancing activation of mTOR (*e*.*g*., specific diets or insulin injection) may be a means of controlling CHIKV infection.

In sum, we have provided evidence for a novel and unexpected mechanism by which CHIKV adapts to mTORC1 inhibition. In the context of acute CHIKV infection, eIF4E is important for the translation of viral proteins, and CHIKV-mediated mTORC1 inhibition increases the phosphorylation of eIF4E, thus favoring viral replication. These data also suggests that targeted engagement of upstream activator of mTORC1 (e.g., Insulin receptor or Akt) or blocking inhibitors of the mTORC1 pathway (e.g., TSC2) may constitute useful strategies for limiting the pathogenesis of acute Chikungunya disease.

## Materials and Methods

### Cells and mice

Wild-type mouse embryonic fibroblasts (MEF) were obtiained from the Korsmeyer laboratory (Farber, Boston, MA, USA). *Atg5* ^*-/-*^ and *atg7* ^*-/-*^ MEF were generous gifts of the Kroemer Laboratory (INSERM U848, Institut Gustave Roussy, Villejuif, France). Primary human foreskin fibroblasts (HFF) were obtained from America Type Culture Collection. All cell lines were mycoplasma free and maintained at 37°C in humidified atmosphere containing 5% CO2 in medium supplemented with 10% heat inactivated fetal calf serum, 100 μg/ml penicillin (Invitrogen); 100 U/ml streptomycin (Invitrogen), and MEM nonessential amino acid (Invitrogen). *irf3* ^*-/-*^ */ irf7* ^*-/-*^ mice were generated by Michael Diamond, with the original strains being provided by Tadatsugu Taniguchi.

### CHIKV infection and drugs treatments in cell lines

The preparation of CHIKV from clinical samples has been previously described (Schuffenecker et al., 2006). CHIKV-21 strain was propagated in C6/36 cells and supernatants were harvested and frozen at -80°C before titration and further use. Recombinant CHIKV expressing GFP under the subgenomic promoter (CHIKV-GFP 5’) was generated using a full-length infection cDNA clone provided by S. Higgs (Vanlandingham et al., 2005). Recombinant CHIKV expressing Luciferase under genomic promoter (CHIKV-Luc.) was a gift from Philippe Despres and was generated as previously described [40]. The CHIKV replicon expressing EGFP (rCHIKV-GFP) was a gift from Gorben Pijlman and was generated as previously describe [41]. MEF or HFF cells (plate at ∼ 50% confluence in 24- or 96-wells plates) were exposed to the indicated viruses for 2h at 37°C, extensively washed with PBS and cultivated for various periods of time in presence of drugs before further analysis. The MOI was defined as the amount of CHIKV infectious units (calculated on BHK cells as PFU) per target cell. In indicated experiments, Rapamycin (100 nM—Calbiochem), TORISEL (0.1 mg/ml—Biovision), PP242 (0.1 μM—Euromedex), 4EGI-1 (25 μM–VWR International), CPG57380 (20 μM–R & D Systems Europe), MNK inhibitor-II (5 μM–Merck Chemicals LTD), PD 0325901 (1 μg/ml–Sigma Aldrich), SB203580 (1 μM–Sigma Aldrich) or LY294002 (25 μM—Ozyme) were used.

### siRNA treatment

Smartpool siRNA targeting *atg5*, *atg7*, *mTOR*, *raptor*, *rictor*, *eIF4E*, *S6K1*, *4EBP-1* and control siRNA were from Dharmacon (Perbio, Berbères, France). MEF or HFF cells (0.1 × 10^6^) were cultured in 6-well plates for 1 day in OptiMEM (Invitrogen) containing 10% FCS and transfected with 30 nM of indicated siRNA using lipofectamine RNAiMAX (Invitrogen). For all experiments, CHIKV infection was performed after 3d siRNA incubation. In all experiments, protein expression of targeted gene was confirmed to be knocked down to <90% of WT expression. Where inducated functional inhibition was evaluated.

### Western blot analysis

Lysates were prepared in 1x Dulbecco’s Phosphate Buffer Saline (DPBS—Invitrogen) containing 1% Nonidet P 40 substitue (NP40 –Signa-Aldrish, MO, USA) and protease inhibitor cocktail (Roche Diagnostics, IN, USA). Total protein was determined by Lowry’s method and 25 mg was loaded on a 4–12% gradient SDS–polyacrylamide gel electrophoresis (Invitrogen). Proteins were transferred to 2 μM nitrocellulose membrane using the Trans-blot turbot kit (Bio-Rad) and blotted over-night with anti-mTOR (rabbit polyclonal, abcam), anti-Rictor (rabbit polyclonal, Cell Signaling), anti-Raptor (rabbit polyclonal, Cell Signaling), anti-Atg5 (mouse monoclonal, Cell Signaling), anti-Atg7 (mouse monoclonal, Cell Signaling), anti-pS6K1 (rabbit polyclonal, Abcam), anti-S6K1 (rabbit polyclonal, Abcam), anti-peIF4E (Ser209) (rabbit polyclonal, Cell Signaling), anti-eIF4E (rabbit polyclonal, Cell Signaling), anti-E1/E2 (rabbit polyclonal, gift from Olivier Schwartz laboratory), anti-C (monoclonal antibody, gift from Olivier Schwartz laboratory), anti-GFP (rabbit polyclonal, Cell Signaling) or anti-GAPDH (rabbit polyclonal, Cell Signaling). Secondary HRP-coupled Abs was detected using ECL Plus (Amersham Pharmacia Biotech).

### Northern blot analysis

MEF were infected with CHIKV-21 at indicated MOI in presence of TORISEL. After 8h and 24h of infection, cells were washed twice in PBS and RNA was TRIzol (Invitrogen) extracted, quantified and diluted to the same concentration. Samples were prepared in NorthernMax formaldehyde loading dye (Ambion) with 1μl of ethidium bromide, heated to 65°C for 10 minutes, then separated on a 1.2% agarose (Lonza) gel containing 1x morpholinepropanesulfonic acid (MOPS), running buffer (Ambion) and 6.7% formaldehyde. RNA was transferred onto nitrocellulose membrane, cross-linked by ultraviolet irradiation (UVP), and prehybridized at 68°C for 1h in ULTRAhyb ultrasensitive hybridization buffer (Ambion). A plasmid used for the expression of CHIKV RNA probes corresponding to the 3′ portion of the E2 glycoprotein was generated by first amplifying the region of the CHIKV genome from 8703 (*5′-GAAGCGACAGACGGGACG-3′*) to 9266 (*5′-GTTACATTTGCCAGCGGAA-3′*) by PCR and subsequently TOPO-TA cloning the PCR product into the pCRTOPO-II vector. RNA probes complementary to positive strand RNA were labeled with ^32^P using the MAXIscript SP6 *In Vitro* Transcription Kit (Ambion), unincorporated nucleotides were removed using illustra MicroSpin S200 HR columns (GE healthcare), and probe was hybridized to the membrane overnight at 68°C. Membranes were washed several times at 68°C with 0.1× SSC with 0.1% SDS, then imaged using Amersham Hyperfilm MP autoradiography film (GE Healthcare).

### CHIKV staining and flow cytometry

MEF or HFF cells were infected with CHIKV-21 or CHIKV-GFP 5’ at the indicated MOI for 24h and fixed with 4% PFA for 20 min. After fixation, cells infected with CHIKV-21 were permeabilized with BD Cytofix/Cytoperm (BD kit, BD Bioscniences) before labeling with anti-E2 (gift form Lecuit lab, Microorganismes et barrières de l’hôte, Institue Pasteur, France). The percentage of E2^+^ or GFP^+^ cells was measured by flow cytometery using FACSCanto (BD Biosciences, MD, USA) and FlowJo software (Tree Star, Inc.).

### Live-cells imaging

MEFs were infected with CHIKV-GFP 5’ in 24-well or 96-well pate and imaged using IncuCyte HD system (Essen BioScience). Frames were captured at 1-hour intervals from 4 separate 950 × 760–μm^2^ regions per well using a 20× objective. Cultures were maintained at 37°C in a Hera cell 240 chamber (Thermo Electron Cormpration) throughout, with all experiments run in triplicate. GFP^+^ cells were counted using IncuCyte ZOOM software (Essen BioScience) and results are represented as green object count per mm^2^. Values from all 4 regions of each well were pooled and averaged across the 3 replicates. Movies were extracted directly from IncuCyte ZOOM software.

### CHIKV titers in cell lines

MEF or HFF cells were infected with CHIKV-21 and supernatants were recovered at 24h after infection. Viral samples were titrated as TCID_50_ endpoint on Vero cells using a standard procedure. Serial 10-fold dilutions (100 μgl) of supernatants were added in six replicates in 96-well plates seeded with 10^4^ Vero cells. The cytopathic effect was scored 5 days after infection and the titers was calculated by determining the last dilution giving 50% of wells with cells displaying a cytopathic effect. Results were expressed as TCID_50_/ml.

### *In vivo* Rapalog treatment, infection and viral titration

*irf3* ^*-/-*^ */ irf7* ^*-/-*^ mice were treated with intra-peritoneal injection of 100 μL solution containing TORISEL (10mg/kg) (*n* = 32) or PBS (*n* = 31) every two days for 8 days. At day 8, mice were infected with 1x10^6^ PFU CHIKV-21 subcutaneously (s.c.) in the bottom chest. For viral titration, skin and muscle were collected after days 1 (*n* = 6 for PBS-treated mice; *n* = 7 for TORISEL-treated mice), 2 (*n* = 5 for PBS-treated mice; *n* = 6 for TORISEL-treated mice), and 3 (*n* = 6 for PBS-treated mice; *n* = 5 for TORISEL-treated mice) of infection, homogenized, and viral samples were titrated as TCID_50_ endpoint on Vero cells using a standard procedure. Clinical score (*n* = 14) was determined at day 2 of infection, based on EAE from K. Racke (*0* = nothing; *1* = limp tail; *2* = mouse don’t grasp the cage with toes but with the ankle; *3* = mouse is unable to return and land on its feet when flipped over; *4* = hindlimb drag behind are not used by the mouse for movement; *5* premoribund stat). Lethality of mice (*n* = 14) was followed for 10 days post-infection.

### Nonradioactive measurements of protein synthesis with SUnSET

SunSet experiments were performed as previously described [23]. To summarize, cells were infected with CHIKV in the presence of TORISEL for 24 hrs. Then cells were cultivated with serum free media containing puromycin (10 υg/ml) for 30 min, washed with PBS and kept in culture for an additional 1h with normal media. Western blot was performed as previously described using an antibody against puromycin (clone 12D10, 1/5000, Millipore).

### Ethics statement

Mouse studies were performed in strict accordance with the Institutional Guiding Principles for Biomedical Research Involving Animals and all experiments were performed in an A3 containment facility. The protocols were approved by the Institutional Committees on Animal Welfare of the Pasteur Institute (OLAW assurance #A5476-01). All efforts were made to minimize suffering.

## Supporting Information

S1 Fig PP242 inhibitor increases CHIKV infection. **(A, B)** MEFs were infected with CHIKV at indicated MOI for 24h in the presence of PP242 (1 μM) and the percentage of E2 positive cells (**A**) or the extracellular viral titers (**B**) were determined. Bars indicate mean values ±SEM from three independent experiments. Student’s test **, P < 0.05.
(PDF)

S2 Fig mTORC2 did not participate in Rapalog-induced enhancement of CHIKV infection. MEFs were pretreated with *raptor* or *rictor* siRNA and infected by CHIKV (MOI = 0.1) in presence of Rapamycin or TORISEL. The percentage of intracellular E2 staining was analyzed by FACS. Bars indicate mean values ±SEM from four independent experiments. Student’s test **, P < 0.05.
(PDF)

S3 Fig The enhancement of mTORC1 activity *via* the inhibition of TSC2 limits CHIKV infection. **(A—C)** MEFs were pretreated with *tsc2* siRNA and Western blot was performed using anti-TSC2 and anti-GAPD antibodies. Additionally, cell lysates were assessed for tyrosine 389 phosphorylation of S6K-1 as a measure of mTORC1 activity (p-S6K1). Similar results were observed in three independent experiments (**A**). MEFs were infected by CHIKV (MOI = 1) and 24h p.i., the percentage of E2 positive cells was measured. Bars indicate mean values ±SEM from three independent experiments **(B)**. MEFs were infected by CHIKV-GFP (MOI = 1) and GFP positive cells were analyzed using real time imaging. Similar results were observed in five independent experiments (**C**). MEFs were infected by CHIKV (MOI = 1) in the presence of Rapamycin (100 nM) and 24h p.i. the percentage of E2 positive cells was determined. Bars indicate mean values ±SEM from three independent experiments **(D)**. Student’s test **, P < 0.05.
(PDF)

S4 Fig Effect of mTORC1 on CHIKV infection is independent of type I IFN pathways. **(A)** Wild type (WT), *irf3* ^*-/-*^ */ irf7* ^*-/-*^ or *ifnar* ^*-/-*^ MEFs were infected with CHIKV (MOI = 0.01) for 24h and stained with an anti-E2 antibody. The percentage of E2 positive cells is shown. Bars indicate mean values ±SEM from five independent experiments. **(B)** MEF cells were infected with CHIKV (MOI = 5) in the presence of TORISEL and Western blot was performed using anti-IκBα, anit-p-p65 and anti-GAPDH antibodies. Similar results were observed in two independent experiments. **(C)** MEFs were infected with CHIKV at indicated MOI in the presence of TORISEL and the concentration of extracellular cytokines were analyzed at 24h post-infection. Bars indicate mean values ±SEM from three independent experiments. **(D, E)** MEFs were infected with CHIKV-GFP (MOI = 1) in presence of TORISEL and/or Actinomycin D (10 ng/ml). **(D)** GFP positive cells were analyzed during 50h of infection using real time imaging. Similar results were observed in three independent experiments. **(E)** Results represent the fold induction of GFP positive cells observed in TORISEL treated cells as compared to untreated cells. Bars indicate mean values ±SEM from three independent experiments. Student’s test **, P < 0.05.
(PDF)

S5 Fig Inhibition of mTORC1 favors CHIKV infection independently of autophagy. (**A-D**) *Atg5* ^-/-^ or *atg7* ^-/-^ MEFs were infected with CHIKV for 24h with indicated MOI in presence of Rapamycin (100 nM) or TORISEL (0.1 mg/ml). The percentage of E2 positive cells (**A**, **C**) or extracellular viral titer (**B**, **D**) are shown. Total viral titers were expressed as TCID_50_/ml. Bars indicate mean values ±SEM from four independent experiments. (**E-G**) WT HFF pre-treated with siRNA specific for *atg5* or *atg7* were infected with CHIKV (MOI = 1) for 24h in presence of Rapamycin or TORISEL. The expression of Atg5, Atg7 and GAPDH was monitored (**E**); the percentage of E2 positive cells was determined by FACS analysis (**F**); and extracellular viral titers were analyzed (**G**). Bars indicate mean values ±SEM from three independent experiments. +, indicates respective siRNA knock-down;-, indicates si-control. Student’s test **, P < 0.05
(PDF)

S6 Fig TORISEL decreases *in vivo* mTORC1 activity. **(A, B)** *Irf3* ^*-/-*^ */ irf7* ^*-/-*^ mice were treated with intra-peritoneal injection of 100 μL of solution containing TORISEL (10 mg/kg) or PBS for 8 days. Skin **(A)** and muscle **(B)** were collected and mTORC1 activity was assessed by following tyrosine 389 phosphorylation of S6K-1 (p-S6K1) by Western blot. Results are representative of three independent experiments. Ø, control; T, TORISEL.
(PDF)

S7 Fig CHIKV infection requires eIF4E activity to replicate. **(A—B)** MEFs were pre-treated with *eif4e* siRNA followed by CHIKV infection (MOI = 1). Western blot using anit-eIF4E and anti-GAPDH was performed and the percentage of E2 positive cells was analyzed at 24hrs p.i. Bars indicate mean values ±SEM from three independent experiments (**A**). MEFs were infected with CHIKV-GFP (MOI = 5) and GFP positive cells were analyzed using real time imaging. Similar results were observed in four independent experiments **(B)**. **(C)** MEFs were infected with CHIKV-GFP (MOI = 5) in presence of 4EGI-1 (4EGI, 10 μM) and GFP positive cells were analyzed using a real time imaging system. Similar results were observed in five independent experiments. **(D)** MEFs were infected with an increasing dose of the CHIKV-Luc recombinant in presence of 4EGI-1 and the luciferase activity was measured 4h p.i. Bars indicate mean values ±SEM from three independent experiments. Student’s test **, P < 0.05.
(PDF)

S8 Fig CHIKV-mediated inhibition of mTOR provides a counter-balance for the antiviral effect of ROS production. **(A, B)** MEF were infected with CHIKV at indicated MOI in the presence of a ROS inhibitor (*N*-acetyl-*L*-cysteine) and/or TORISEL. The percentage of E2 staining expressing cells was determined by FACS analysis. Bar indicate mean values ±SEM from four independent experiments. **(C)** MEFs were pre-treated with *mtor* siRNA followed by CHIKV infection at indicated MOI. The percentage of intracellular E2 staining was analyzed as described in A. Error bar indicate mean values ±SEM from four independent experiments. Student’s test **, P < 0.05.
(PDF)

S1 Movie (Related to Fig 4) mTORC1 inhibitors enhance translation of both structural and non-structural proteins of CHIKV. MEFs were infected with CHIKV-GFP (MOI = 1) in the absence of TORISEL and GFP staining was followed using real time imaging.
(MOV)

S2 Movie (Related to Fig 4) mTORC1 inhibitors enhance translation of both structural and non-structural proteins of CHIKV. MEFs were infected with CHIKV-GFP (MOI = 1) in the presence of TORISEL and GFP staining was followed using real time imaging.
(MOV)
