# Supplementary figures and images for: Inhibition of mTORC1 Enhances the Translation of Chikungunya Proteins via the Activation of the MnK/eIF4E Pathway
Source: PLoS Pathog. 2015 Aug 28;11(8):e1005091. doi: 10.1371/journal.ppat.1005091 (PMC4552638; doi:10.1371/journal.ppat.1005091)

A.

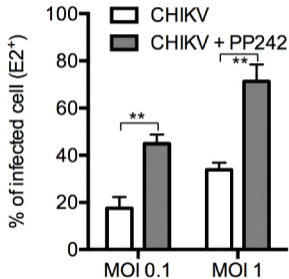

B.

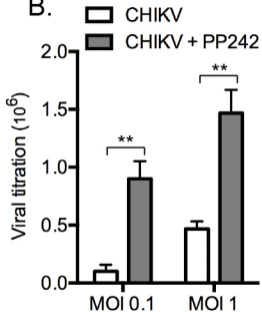

Supplement: S1 Fig — (A, B) MEFs were infected with CHIKV at indicated MOI for 24h in the presence of PP242 (1 μM) and the percentage of E2 positive cells (A) or the extracellular viral titers (B) were determined. Bars indicate mean values ±SEM from three independent experiments. Student’s test **, P < 0.05. (PDF) [file ppat.1005091.s001.pdf]

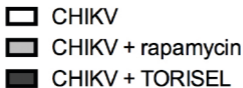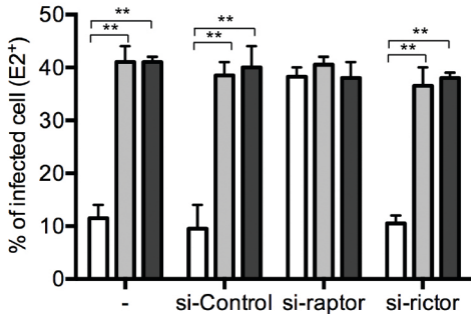

Supplement: S2 Fig — MEFs were pretreated with raptor or rictor siRNA and infected by CHIKV (MOI = 0.1) in presence of Rapamycin or TORISEL. The percentage of intracellular E2 staining was analyzed by FACS. Bars indicate mean values ±SEM from four independent experiments. Student’s test **, P < 0.05. (PDF) [file ppat.1005091.s002.pdf]

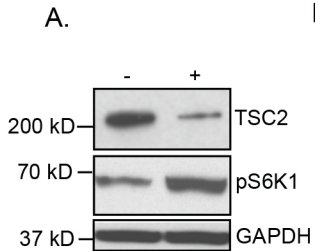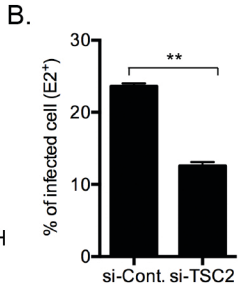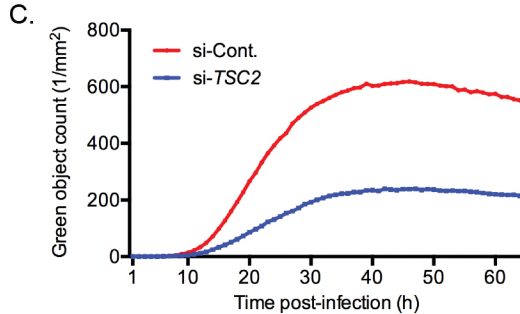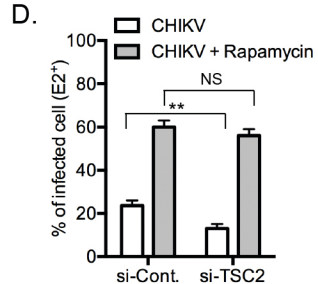

Supplement: S3 Fig — (A—C) MEFs were pretreated with tsc2 siRNA and Western blot was performed using anti-TSC2 and anti-GAPD antibodies. Additionally, cell lysates were assessed for tyrosine 389 phosphorylation of S6K-1 as a measure of mTORC1 activity (p-S6K1). Similar results were observed in three independent experiments (A). MEFs were infected by CHIKV (MOI = 1) and 24h p.i., the percentage of E2 positive cells was measured. Bars indicate mean values ±SEM from three independent experiments (B). MEFs were infected by CHIKV-GFP (MOI = 1) and GFP positive cells were analyzed using real time imaging. Similar results were observed in five independent experiments (C). MEFs were infected by CHIKV (MOI = 1) in the presence of Rapamycin (100 nM) and 24h p.i. the percentage of E2 positive cells was determined. Bars indicate mean values ±SEM from three independent experiments (D). Student’s test **, P < 0.05. (PDF) [file ppat.1005091.s003.pdf]

A.

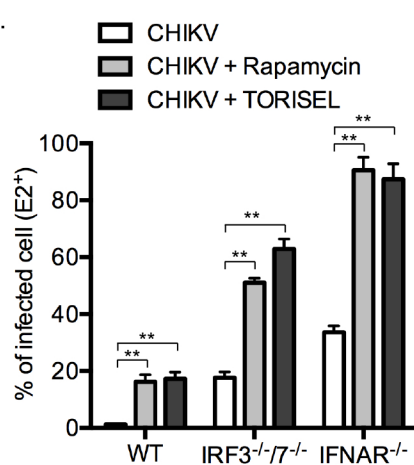

B.

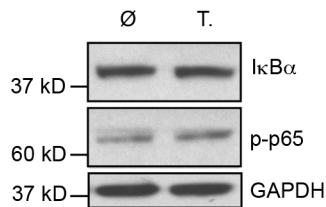

C.

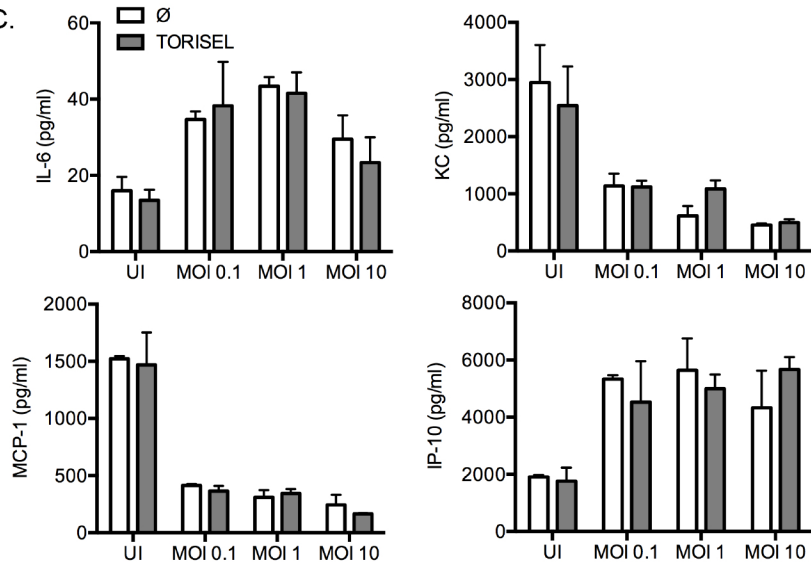

D.

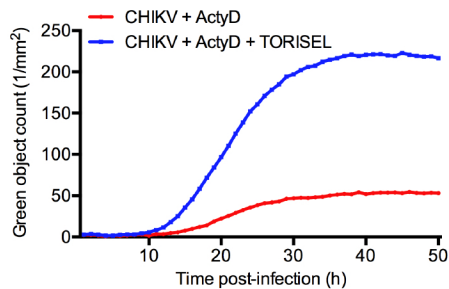

E.

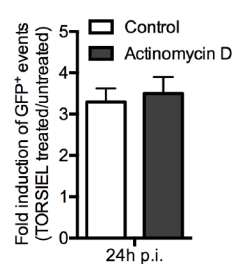

Supplement: S4 Fig — (A) Wild type (WT), irf3 -/- / irf7 -/- or ifnar -/- MEFs were infected with CHIKV (MOI = 0.01) for 24h and stained with an anti-E2 antibody. The percentage of E2 positive cells is shown. Bars indicate mean values ±SEM from five independent experiments. (B) MEF cells were infected with CHIKV (MOI = 5) in the presence of TORISEL and Western blot was performed using anti-IκBα, anit-p-p65 and anti-GAPDH antibodies. Similar results were observed in two independent experiments. (C) MEFs were infected with CHIKV at indicated MOI in the presence of TORISEL and the concentration of extracellular cytokines were analyzed at 24h post-infection. Bars indicate mean values ±SEM from three independent experiments. (D, E) MEFs were infected with CHIKV-GFP (MOI = 1) in presence of TORISEL and/or Actinomycin D (10 ng/ml). (D) GFP positive cells were analyzed during 50h of infection using real time imaging. Similar results were observed in three independent experiments. (E) Results represent the fold induction of GFP positive cells observed in TORISEL treated cells as compared to untreated cells. Bars indicate mean values ±SEM from three independent experiments. Student’s test **, P < 0.05. (PDF) [file ppat.1005091.s004.pdf]

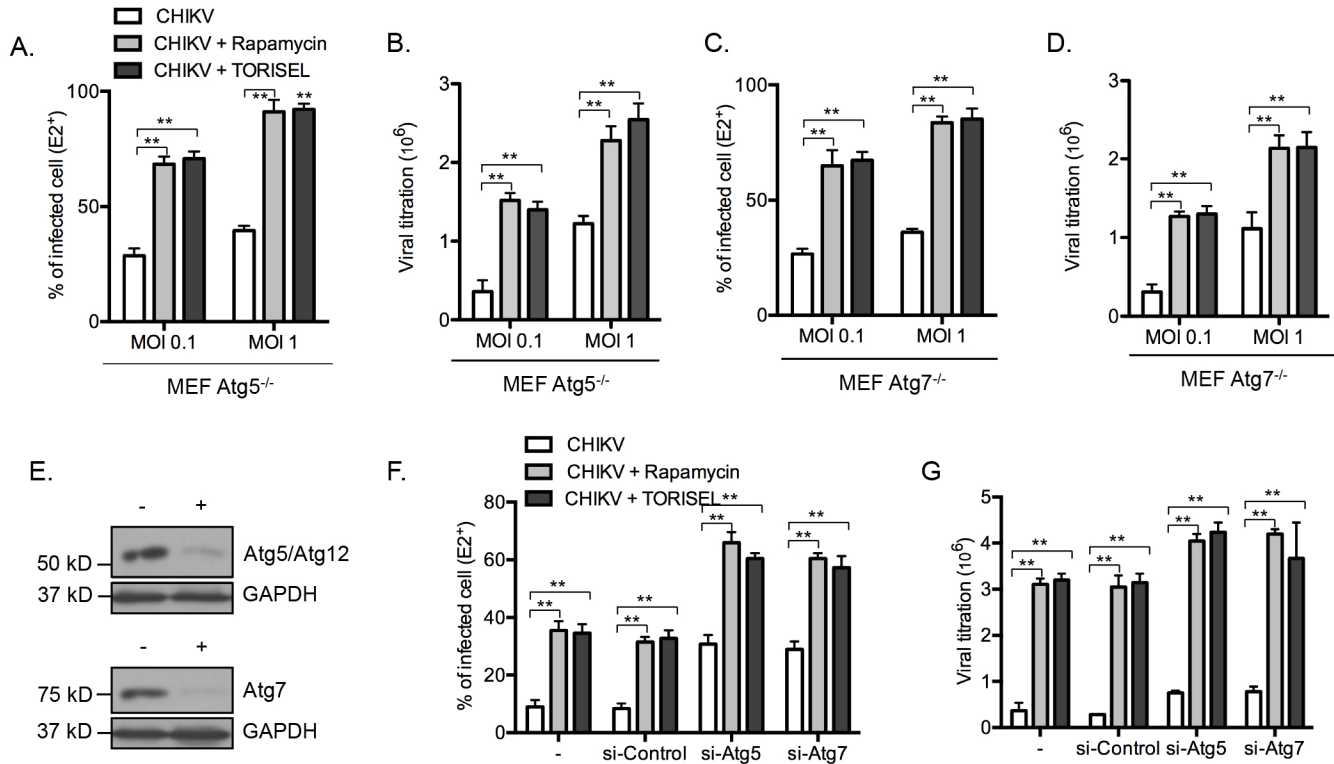

Supplement: S5 Fig — (A-D) Atg5 -/- or atg7 -/- MEFs were infected with CHIKV for 24h with indicated MOI in presence of Rapamycin (100 nM) or TORISEL (0.1 mg/ml). The percentage of E2 positive cells (A, C) or extracellular viral titer (B, D) are shown. Total viral titers were expressed as TCID50/ml. Bars indicate mean values ±SEM from four independent experiments. (E-G) WT HFF pre-treated with siRNA specific for atg5 or atg7 were infected with CHIKV (MOI = 1) for 24h in presence of Rapamycin or TORISEL. The expression of Atg5, Atg7 and GAPDH was monitored (E); the percentage of E2 positive cells was determined by FACS analysis (F); and extracellular viral titers were analyzed (G). Bars indicate mean values ±SEM from three independent experiments. +, indicates respective siRNA knock-down;-, indicates si-control. Student’s test **, P < 0.05 (PDF) [file ppat.1005091.s005.pdf]

A.

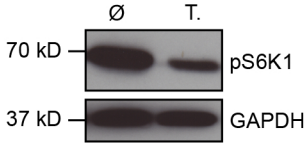

B.

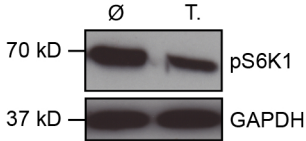

Supplement: S6 Fig — (A, B) Irf3 -/- / irf7 -/- mice were treated with intra-peritoneal injection of 100 μL of solution containing TORISEL (10 mg/kg) or PBS for 8 days. Skin (A) and muscle (B) were collected and mTORC1 activity was assessed by following tyrosine 389 phosphorylation of S6K-1 (p-S6K1) by Western blot. Results are representative of three independent experiments. Ø, control; T, TORISEL. (PDF) [file ppat.1005091.s006.pdf]

A.

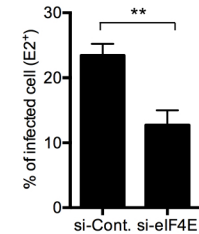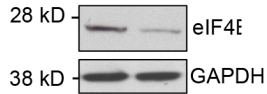

B.

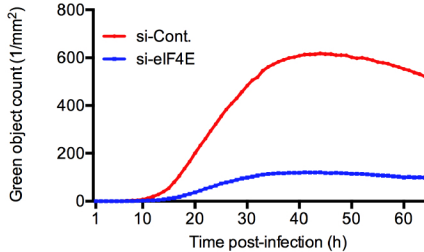

C.

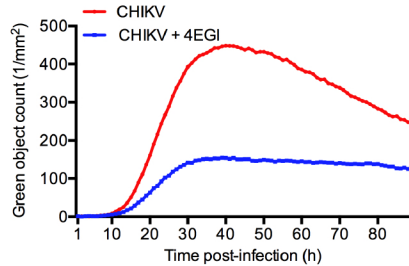

D.

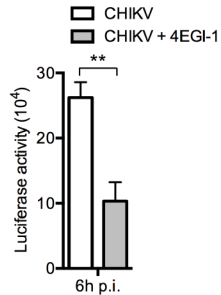

Supplement: S7 Fig — (A—B) MEFs were pre-treated with eif4e siRNA followed by CHIKV infection (MOI = 1). Western blot using anit-eIF4E and anti-GAPDH was performed and the percentage of E2 positive cells was analyzed at 24hrs p.i. Bars indicate mean values ±SEM from three independent experiments (A). MEFs were infected with CHIKV-GFP (MOI = 5) and GFP positive cells were analyzed using real time imaging. Similar results were observed in four independent experiments (B). (C) MEFs were infected with CHIKV-GFP (MOI = 5) in presence of 4EGI-1 (4EGI, 10 μM) and GFP positive cells were analyzed using a real time imaging system. Similar results were observed in five independent experiments. (D) MEFs were infected with an increasing dose of the CHIKV-Luc recombinant in presence of 4EGI-1 and the luciferase activity was measured 4h p.i. Bars indicate mean values ±SEM from three independent experiments. Student’s test **, P < 0.05. (PDF) [file ppat.1005091.s007.pdf]

A.

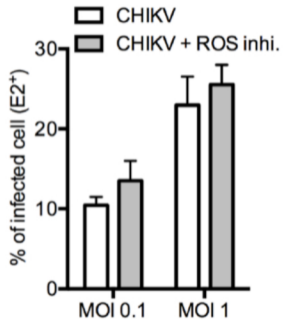

B.

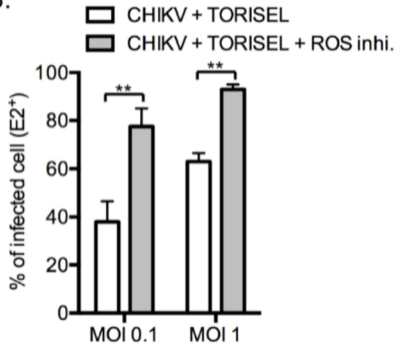

C.

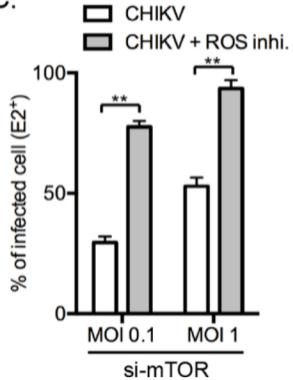

Supplement: S8 Fig — (A, B) MEF were infected with CHIKV at indicated MOI in the presence of a ROS inhibitor (N-acetyl-L-cysteine) and/or TORISEL. The percentage of E2 staining expressing cells was determined by FACS analysis. Bar indicate mean values ±SEM from four independent experiments. (C) MEFs were pre-treated with mtor siRNA followed by CHIKV infection at indicated MOI. The percentage of intracellular E2 staining was analyzed as described in A. Error bar indicate mean values ±SEM from four independent experiments. Student’s test **, P < 0.05. (PDF) [file ppat.1005091.s008.pdf]
